# Beyond oxidative stress: Ferroptosis as a novel orchestrator in neurodegenerative disorders

**DOI:** 10.3389/fimmu.2025.1683876

**Published:** 2025-12-05

**Authors:** Yaqiao Yi, Pu Jia, Peipei Xie, Xiru Peng, Xuan Zhu, Shuting Yin, Yanfang Luo, Ying Deng, Lifei Wan

**Affiliations:** 1 Hunan University of Chinese Medicine, Changsha, China; 2 Department of Nephrology, The Central Hospital of Shaoyang, Shaoyang, Hunan, China; 3 People’s Hospital of Ningxiang City, Ningxiang, China

**Keywords:** ferroptosis, iron metabolism, lipid peroxidation, neuroinflammation, neurodegenerative diseases, brain disorders

## Abstract

Neurodegenerative diseases are a group of disorders characterized by progressive loss of neuronal function due to degenerative damage to neural cells. Ferroptosis, a newly identified form of regulated cell death, is pathologically defined by iron-dependent accumulation of lipid peroxides, mitochondrial shrinkage, and increased mitochondrial membrane density. Unlike apoptosis or necrosis, ferroptosis is driven by a combination of factors, including excessive lipid peroxidation, disruption of iron homeostasis, and depletion of antioxidant defenses such as glutathione (GSH) and glutathione peroxidase 4 (GPX4). The ferroptotic process engages multiple biological functions—such as iron metabolism, lipid metabolism, oxidative stress, mevalonate signaling, transsulfuration pathways, heat shock protein activation, glutamate/cystine transport, and GSH biosynthesis. While initial studies focused on its role in cancer, accumulating evidence now links ferroptosis to neurological disorders. Ferroptosis has been implicated in the pathophysiology of stroke, traumatic brain injury, and major neurodegenerative diseases such as Alzheimer’s disease (AD), Parkinson’s disease (PD), and Huntington’s disease (HD). Several small-molecule inhibitors—including ferrostatin-1, liproxstatin-1, and iron chelators such as deferoxamine (DFO)—have demonstrated efficacy in animal models by attenuating neuronal damage and improving behavioral outcomes through the suppression of ferroptosis. In addition, natural compounds have emerged as promising candidates for targeting ferroptosis due to their structural diversity, low toxicity, and multitarget regulatory properties. These agents offer potential leads for developing novel neuroprotective therapeutics. Neurodegenerative diseases remain a significant global health burden, with limited effective treatments available to date. Modulation of ferroptosis presents a new conceptual framework for therapeutic intervention, offering hope for disease-modifying strategies. This review summarizes recent advances in understanding the role of ferroptosis in neurodegenerative disease mechanisms, focusing on its contribution to pathological progression, molecular regulation, and therapeutic interventions. By integrating current findings, we aim to provide theoretical insights into novel pathogenic mechanisms and scientific guidance for the development of targeted therapies that modulate ferroptosis to slow or halt disease progression.

## 1 Introduction

Neurodegenerative diseases are irreversible disorders of the nervous system caused by progressive loss of neurons in the brain and spinal cord (1). These diseases result from the degeneration of neurons and/or their myelin sheaths, leading to worsening functional impairments over time (2). Neurodegenerative diseases can be classified as acute or chronic. Acute neurodegeneration includes conditions such as cerebral ischemia (CI), brain injury (BI), and epilepsy, whereas chronic forms encompass major diseases like Alzheimer’s disease (AD) and Parkinson’s disease (PD). Among these, AD and PD are the two most prevalent neurodegenerative diseases in the elderly population—AD is primarily characterized by cognitive decline, while PD manifests with motor dysfunction (3). Despite extensive research, the exact etiology of most neurodegenerative diseases remains elusive, and no curative treatments currently exist, making these diseases a significant threat to human health and quality of life. Age-associated neurodegenerative diseases pose an increasing risk in the context of global population aging (2–4). According to the Global Burden of Disease (GBD) study, in 2019 alone, the global prevalence of AD and related dementias reached 51.62 million, with an overall prevalence rate of 667.2 per 100,000 individuals and an age-standardized prevalence of 682.5 per 100,000 (5). In China, the incidence of PD among individuals aged ≥65 years is approximately 1.7%, with approximately 100,000 new cases reported annually, reflecting a rapidly rising trend. More than 2.5 million people in China suffer from AD, making it the country with both the largest absolute number of patients and the fastest-growing AD population globally (6). Therefore, there is an urgent need to uncover the underlying mechanisms of neurodegenerative diseases.

In recent decades, studies have identified several key factors contributing to neurodegeneration, including inflammation, aging, oxidative stress, and genetic mutations (7, 8). Given that neurodegenerative diseases predominantly affect middle-aged and elderly populations, cellular senescence has been increasingly recognized as a central driver of disease onset and progression. Aging-related accumulation of senescent cells in the nervous system leads to neuronal dysfunction and exacerbates disease pathology (9), highlighting cellular aging as one of the most critical risk factors for neurodegeneration (4). Evidence suggests that cellular stress responses, such as oxidative damage and mitochondrial dysfunction, accelerate cellular aging (10). During physiological aging and the development of age-related pathologies, the cellular response to oxidative stress and its capacity for repair become dysregulated, further promoting senescence and cell death (11).

Over the past few decades, multiple modes of cell death have been defined. The Nomenclature Committee on Cell Death (NCCD) classifies cell death into two broad categories based on functional and mechanistic criteria: accidental cell death (ACD), a passive process, and regulated cell death (RCD), an active, gene-controlled mechanism (12). RCD encompasses several subtypes, among which apoptosis—the caspase-dependent form of programmed cell death—is the most extensively studied (12). However, in pathological contexts such as infection, trauma, and neurodegenerative diseases, alternative non-apoptotic forms of RCD dominate, including necroptosis, pyroptosis, autophagy, and ferroptosis (13–16).

Ferroptosis, first identified and described by Dixon et al. in 2012, is a unique form of regulated necrosis driven by iron-dependent accumulation of toxic reactive oxygen species (ROS) within lipid membranes (17). Subsequent studies revealed that ferroptosis may serve as a crucial cell death mechanism in acute brain injuries such as ischemic stroke and intracerebral hemorrhage (18). At the molecular level, ferroptosis is characterized by iron-dependent lipid ROS accumulation, which results from irreversible lipid peroxidation and membrane permeabilization (19). Morphologically, ferroptosis presents with distinct mitochondrial changes, including organelle shrinkage and loss of cristae (20). Although the precise mechanisms driving these morphological alterations remain unclear, they serve as hallmark indicators of ferroptosis. The concept of iron-dependent cell death is not entirely novel. Prior to the formal identification of ferroptosis, numerous studies had already linked iron overload and oxidative stress to pathological cell death in neurodegenerative diseases and brain injury (21–23). Accumulating evidence indicates that many neurodegenerative diseases feature localized iron accumulation in specific regions of the central or peripheral nervous system. This iron deposition often results from altered cellular iron distribution, triggering Fenton chemistry and lipid peroxidation—processes tightly associated with the progression of neurological disease and accompanied by reductions in GSH and glutathione peroxidase 4 (GPX4) levels. Currently, therapeutic strategies targeting ferroptosis are rapidly evolving, including iron chelation, enhancement of antioxidant defenses, lipid metabolism modulation, and the development of specific small-molecule inhibitors. These approaches have demonstrated neuroprotective effects in various preclinical models, laying a foundation for future clinical translation.

However, the precise role of ferroptosis across different neurodegenerative conditions remains incompletely understood. A major challenge lies in developing targeted interventions that modulate ferroptosis without compromising essential physiological functions. To address this, this review summarizes recent advances in ferroptosis mechanisms and their roles in neurodegenerative diseases. Looking ahead, emerging technologies such as single-cell multi-omics, spatial transcriptomics, and organoid modeling offer unprecedented opportunities to dissect the complex regulatory networks linking ferroptosis to neurodegeneration. Ultimately, targeting ferroptosis may open new avenues for early diagnosis and precision therapy in neurodegenerative diseases, advancing the field toward more effective and individualized treatment paradigms.

## 2 Mechanisms of neurodegenerative diseases

Neurodegenerative diseases are a group of age-related disorders characterized by highly heterogeneous pathogenesis and clinical manifestations, with progressive loss of vulnerable neurons and gradual decline in brain function (24). As irreversible disorders caused by the loss of neurons in the brain and spinal cord, recent studies have identified eight hallmarks of neurodegeneration: pathological protein aggregation, synaptic and neuronal network dysfunction, proteostasis imbalance, cytoskeletal abnormalities, altered energy homeostasis, DNA and RNA damage, inflammation, and neuronal cell death (25, 26). These findings highlight the necessity for multitargeted and personalized therapeutic approaches, offering new directions for drug discovery. Although these diseases exhibit distinct histopathological features, they may share common cellular and molecular mechanisms. Thus, identifying effective preventive strategies is crucial for the treatment of neurodegenerative diseases.

### 2.1 Genetic factors in neurodegenerative diseases

Age remains a key risk factor for AD, which predominantly affects the elderly and causes significant burdens on daily living through progressive memory and learning deficits (4). Approximately 4%–8% of AD cases are influenced by genetic factors, leading to early-onset familial Alzheimer’s disease (FAD) driven by mutations in genes such as APP and PSEN1 (25). These genes are involved in lipid peroxidation and ferroptosis. For example, ALDH2 overexpression protects against APP/PSEN1 mutation-induced cardiac atrophy, contractile dysfunction, and mitochondrial damage by regulating PSEN1–ACSL4-mediated lipid peroxidation and ferroptosis (25, 26). PD is characterized by reduced dopamine levels and the presence of Lewy bodies. Studies show that single-gene mutations play a critical role in familial forms of PD. For instance, mutations in genes such as DJ-1 accelerate disease progression. In addition, patients with PD exhibit elevated lipid peroxidation, glutathione depletion, deficiencies in DJ-1 and coenzyme Q10, reduced glutathione peroxidase 4 activity, mitochondrial dysfunction, iron accumulation, and α-synuclein aggregation. Together, these features are closely associated with ferroptosis, indicating that ferroptosis contributes to the pathogenesis of PD (27, 28).

Furthermore, recent studies have shown that under stress from ferroptosis inducers, DJ-1 can significantly suppress ferroptosis. This protective effect occurs through the preservation of S-adenosylhomocysteine hydrolase activity, which helps inhibit ferroptosis (28, 29).

### 2.2 Mitochondrial dysfunction in neurodegenerative pathology

Mitochondria are central regulators of cellular respiration, metabolism, energy production, intracellular signaling, and free radical generation (10, 30). Accumulating evidence indicates that mitochondrial dysfunction plays a pivotal role in regulating cell death and aging, serving as a hallmark of neurodegeneration (31). In the central nervous system (CNS), the sufficient energy supply required for neuronal survival and excitability largely depends on mitochondrial function (32). In AD, mitochondrial defects represent one of the earliest and most recognized pathological features, particularly linked to impaired energy metabolism (33). Targeting mitochondrial pathways has shown promise in mitigating neurodegeneration and functional impairment, indicating that mitochondrial disruption accelerates AD progression. In Parkinson’s disease, mitochondrial dysfunction also plays a central role (34). Both familial and sporadic forms of PD involve mutations in mitochondrial-associated proteins—such as PINK1, Parkin, DJ-1, and α-synuclein. One of the most compelling pieces of evidence is the observed reduction in mitochondrial complex I activity in postmortem brains of PD patients, reinforcing mitochondrial involvement in PD pathology. This suggests that improving mitochondrial function may serve as a key strategy for slowing disease progression. In amyotrophic lateral sclerosis (ALS), mitochondrial impairments—including reduced ATP synthesis, altered axonal distribution, morphological changes, elevated ROS production, and respiratory chain defects—are associated with rapid disease progression (35). Morphological and biochemical mitochondrial abnormalities have also been confirmed in postmortem spinal cords of ALS patients, further supporting the central role of mitochondrial dysfunction in ALS pathogenesis (36). Similarly, in Huntington’s disease (HD), mitochondrial dysfunction leads to neuronal degeneration, contributing to disease onset and progression.

### 2.3 Relationship between oxidative stress and neurodegenerative disorders

With aging, redox imbalance develops alongside elevated levels of ROS and reactive nitrogen species (RNS). These changes contribute to regional neuronal dysfunction, memory impairment, and cognitive decline. Such findings support a close link between oxidative damage and neuronal injury (37, 38). AD, the most common neurodegenerative disorder among the elderly, involves multiple molecular events in its pathogenesis, with hallmark features including neurofibrillary tangles (NFTs), senile plaques, and neuronal loss (39, 40). Several lines of evidence suggest that oxidative stress is a key driver of AD progression. In AD mouse models, peroxidase activity was shown to improve cognition and spatial memory while reducing Aβ plaque deposition in the cortex and hippocampus (41). In other neurodegenerative diseases, Parkinson’s disease is the second most prevalent neurodegenerative disorder among individuals over 60 years of age (42–44). Dopaminergic neurons are especially susceptible to ROS-induced damage (45). The neurotoxin MPP^+^, a metabolite transported into dopaminergic neurons via the dopamine transporter, accumulates in the mitochondria and inhibits complex I activity of the electron transport chain, reducing ATP production and increasing ROS release, ultimately causing degeneration of dopaminergic neurons in the substantia nigra and striatum (46). Studies on PD mouse models have also demonstrated that DJ-1 plays a role in antioxidant defense (47). Mutations in several genes—including SOD1, TARDBP, FUS, UBQLN2, C9orf72, and TAF15—have been found in approximately 5% of ALS cases. Mutant SOD1 enhances ROS/RNS production, accelerating disease progression (48, 49). Huntington’s disease is considered a protein misfolding disorder, where excessive ROS production leads to abnormal protein folding and impaired neurotransmission. HD is also associated with disrupted mitochondrial morphology and function, resulting in energy deficiency and progressive neurodegeneration. Animal models of HD have revealed that when antioxidant capacity is compromised, mitochondrial function deteriorates (50), highlighting the role of oxidative stress in HD pathogenesis.

### 2.4 Relationship between aging and neurodegenerative diseases

Given the high prevalence of neurodegenerative diseases in older adults, aging is considered the primary risk factor for neurodegeneration (51). Due to the high metabolic and energetic demands of the brain, neurons are particularly prone to ROS overproduction, which damages brain tissue. This damage increases with age due to declining DNA repair capacity, emphasizing the importance of understanding aging mechanisms in developing interventions for neurodegenerative diseases (52). Currently, the most common neurodegenerative diseases—such as AD and PD—predominantly affect the elderly population. Cellular senescence in AD may be driven by increased DNA damage and reduced DNA repair capacity, which worsens with disease progression (53). Similarly, PD pathogenesis involves multiple pathways, including α-synuclein aggregation, mitochondrial dysfunction, oxidative stress, calcium dysregulation, axonal transport defects, and neuroinflammation. At the cellular level, increased α-synuclein deposition correlates with cellular aging, evidenced by elevated numbers of senescent cells and increased β-galactosidase expression in postmortem PD brain tissues (54).

Beyond AD and PD, numerous other neurodegenerative diseases show strong associations with cellular senescence. For example, ALS presents both sporadic and familial forms, often linked to defective DNA repair, which leads to neuronal damage, premature aging, and subsequent neurodegeneration (55). Clinical evidence supports the view that DNA repair deficiencies drive neuronal aging and degeneration in ALS. In HD, age-related telomere attrition impairs transcriptional regulation, a process thought to underlie widespread neuronal death in HD (56). These observations reinforce the concept that aging is deeply intertwined with neurodegenerative processes. Therefore, future research should focus on the intersection between aging and neurodegeneration, potentially informing novel therapeutic strategies for these devastating conditions.

### 2.5 Ferroptosis in neurodegenerative pathogenesis

**Figure 1:**
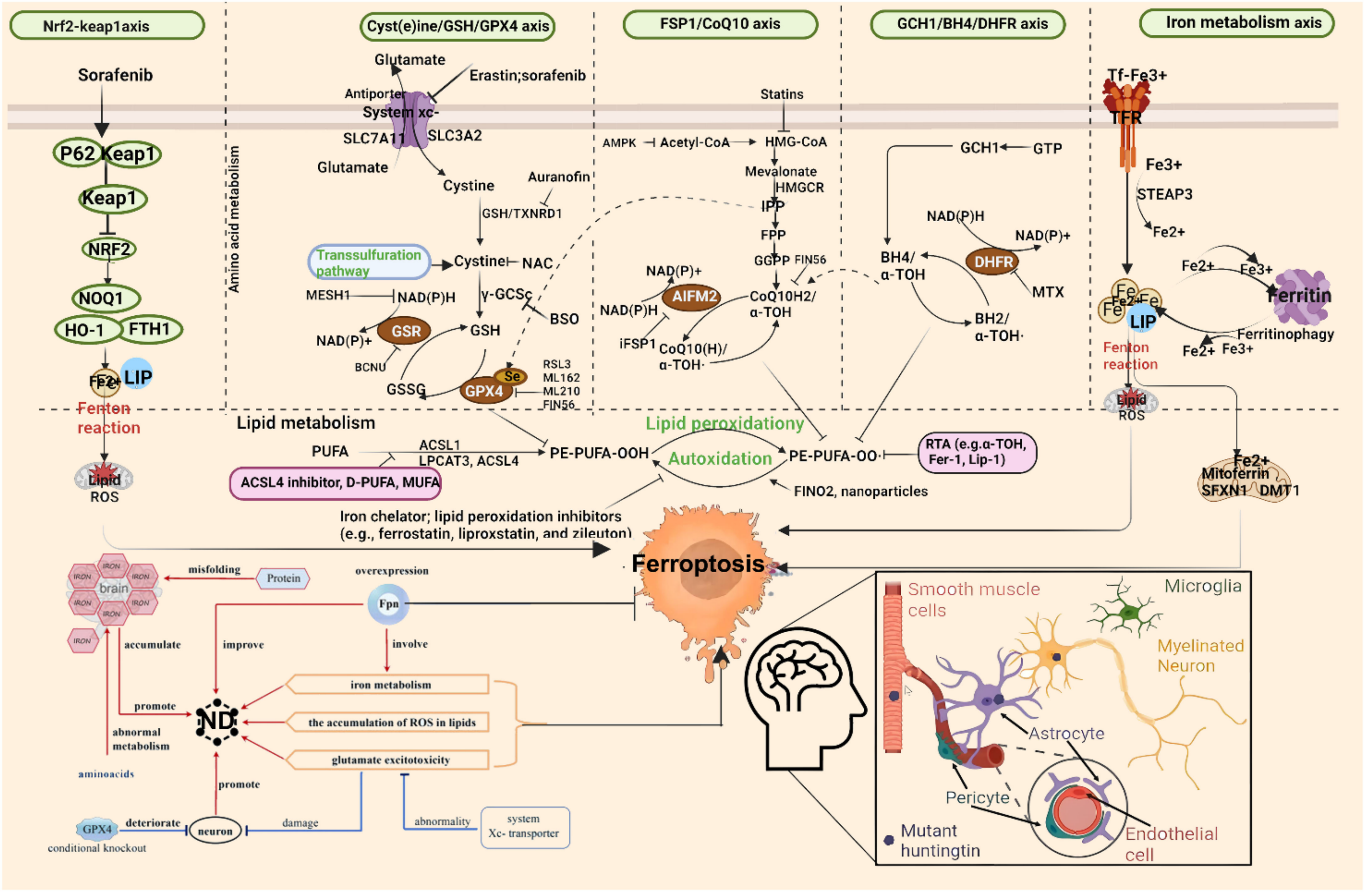
Molecular mechanisms of ferroptosis in neurodegenerative diseases. From left to right: The diagram illustrates ferroptosis mechanisms induced by distinct regulatory pathways. The classical regulatory pathway is shown in the first column on the left: cystine is imported into the cell via the system Xc^−^ and subsequently reduced to cysteine through either the GSH-dependent or TXNRD1-dependent pathway, promoting GSH synthesis. GSR catalyzes the reduction of GSSG to GSH using electrons provided by NADPH/H^+^. The second column highlights the FSP1/CoQ10 axis. This pathway has been shown to protect cells from ferroptosis induced by either pharmacological inhibition or genetic deletion of GPX4. Unlike the GPX4/GSH pathway, FSP1 exerts its protective effect through coenzyme Q10 (CoQ10) and α-tocopherol. The rightmost column summarizes additional mechanisms involved in ferroptosis suppression. GPX4, glutathione peroxidase 4; GSH, glutathione; TXNRD1, thioredoxin reductase 1; GSR, glutathione reductase; GSSG, oxidized glutathione; FSP1, ferroptosis suppressor protein 1; SLC7A11, solute carrier family 7 member 11; SLC40A1, solute carrier family 40 member 1.

Iron is the most abundant transition metal in the human brain and participates in vital CNS functions such as mitochondrial energy transduction, enzymatic catalysis, myelination, synaptic plasticity, and neurotransmitter synthesis and degradation (57). Since the formal identification of ferroptosis, researchers have increasingly viewed it as a critical contributor to neuronal death in PD, AD, and HD (58). Animal models and anatomical studies in humans reveal varying degrees of iron accumulation in the aging brain, which may lead to age-dependent ferroptotic cell death. Long-term exposure to excess iron in mice disrupts membrane transporter function and intracellular iron homeostasis, elevating ROS and malondialdehyde (MDA) levels, ultimately triggering neuronal death (59). These findings underscore the critical role of cerebral iron content in linking neurodegenerative diseases and ferroptosis. For instance, Alzheimer’s disease, the most prevalent neurodegenerative disorder, is characterized by NFTs. Studies have reported correlations between brain iron and ferritin levels and the extent of amyloid-beta deposition (60). These observations suggest that ferroptosis may play a central role in AD pathogenesis, warranting deeper investigation. In Parkinson’s disease, neuropathological hallmarks include the loss of catecholaminergic neurons in the substantia nigra and locus coeruleus. Clinically, iron levels in the substantia nigra are significantly higher in PD patients compared to controls (61). Moreover, selective depletion of glutathione (GSH) is a defining feature of PD, aligning with the core characteristics of ferroptosis (62) and implicating ferroptosis as a potential driver of dopaminergic neuron loss. In Huntington’s disease, GSH dysregulation, iron overload, and lipid peroxidation are commonly observed in HD patients. However, administration of the ferroptosis inhibitor ferrostatin-1 has been shown to suppress lipid peroxidation, restore GSH levels, reduce iron accumulation, and markedly attenuate neuronal death (63), suggesting that targeting ferroptosis could provide therapeutic benefits in HD. The mechanism of ferroptosis in neurodegenerative diseases is summarized in **Figure 1**.

Despite significant challenges in managing neurodegenerative diseases, advances in mechanistic understanding are rapidly emerging. As outlined above, multiple interconnected mechanisms—including genetic predispositions, mitochondrial dysfunction, oxidative stress, aging, and ferroptosis—converge in driving neurodegenerative pathology. However, our comprehension of the full complexity underlying these diseases remains incomplete. Beyond the discussed mechanisms, neurodegeneration may also involve inflammatory responses, energy metabolism defects, endoplasmic reticulum stress, immune dysfunction, and viral infection. Further in-depth exploration of these interwoven mechanisms, supported by modern experimental technologies and multidisciplinary integration, will be essential to uncover robust strategies for the prevention and treatment of neurodegenerative diseases. Such efforts hold the potential to transform current therapeutic paradigms, offering hope for precision medicine in the fight against neurodegeneration.

## 3 Ferroptosis: characteristics and molecular mechanisms

In recent years, ferroptosis, a newly identified form of regulated cell death, has emerged as a distinct mechanism characterized by iron-dependent lipid peroxidation and morphologically distinct from other modes of cell death (64, 65). Ferroptosis is closely associated with various human diseases, including cancer, aging-related disorders, neurodegenerative diseases, and ischemia–reperfusion injury (66–68). For instance, ferroptosis inducers such as sulfasalazine and erastin have been shown to significantly inhibit tumor cell proliferation (69), highlighting their therapeutic potential. 1) Morphological features of ferroptosis: The morphological hallmarks of ferroptosis include loss of plasma membrane integrity, cytoplasmic and organelle swelling, chromatin condensation, and increased cellular detachment and aggregation (70). Notably, the mitochondria exhibit characteristic changes such as shrinkage, increased membrane density, reduction or disappearance of cristae, and outer membrane rupture (71). These structural alterations differentiate ferroptosis from other types of cell death such as apoptosis or necrosis. 2) Biochemical features of ferroptosis: Ferroptosis is biochemically defined by the accumulation of ROS, iron overload, and lipid peroxidation (72). Under normal conditions, cells maintain redox balance through antioxidant systems. However, when these defenses are compromised, excess Fe²^+^ mediates Fenton reactions that generate hydroxyl radicals—highly reactive species that oxidize polyunsaturated fatty acids (PUFAs) in phospholipids. This oxidative damage destabilizes the lipid bilayer, leading to membrane breakdown and ultimately triggering ferroptosis (72). Differences between ferroptosis and other types of programmed cell death are shown in **Table 1**.

**Table 1:** Comparison of several programmed cell deaths.

Feature	Ferroptosis	Apoptosis	Autophagy	Necroptosis
Morphological characteristics	Mitochondrial shrinkage, increased bilayer membrane density, decreased or vanished cristae, rupture of the outer mitochondrial membrane	Cell and nuclear shrinkage, chromatin condensation, nuclear fragmentation, formation of apoptotic bodies, cytoskeleton disassembly	Formation of numerous autophagosomes	Cell and organelle swelling, chromatin condensation, plasma membrane rupture, leakage of intracellular contents
Biochemical characteristics	Iron accumulation, lipid peroxidation	Caspase activation, DNA fragmentation	Conversion of LC3-I to LC3-II, degradation of autophagic substrates (e.g., p62)	Decreased ATP levels, activation of RIP1 and MLKL
Immunological characteristics	Release of DAMPs, pro-inflammatory	Usually does not trigger inflammation, but delayed clearance can expose and release DAMPs, leading to immune responses	Usually suppresses inflammation, but failed clearance or excessive activation may induce inflammation	Typically releases DAMPs, causing inflammatory responses
Key proteins	GPX4, TFR1, ferritin, SLC7A11, NRF2, P53, ACSL4, FSP1	Caspase, Bcl-2, Bax, P53, Fas	ATG5, ATG7, LC3, Beclin-1, DRAM3, TFEB	RIP1, RIP3

### 3.1 Molecular mechanisms of ferroptosis

Ferroptosis is intricately linked to intracellular metabolism involving amino acids, lipids, and iron. Additional metabolic pathways and regulatory factors also influence cellular susceptibility to ferroptosis.

#### 3.1.1 Relationship between amino acid metabolism and ferroptosis

The system Xc^−^, a cystine/glutamate antiporter located on the cell membrane, consists of the light chain SLC7A11 and heavy chain SLC3A2, linked via disulfide bonds. This system mediates a 1:1 exchange of extracellular cystine for intracellular glutamate, enabling cystine uptake and subsequent conversion into L-cysteine, which is used for the synthesis of GSH (73). GPX4 serves as a key enzyme in the antioxidant defense system. Utilizing GSH as a cofactor, GPX4 catalyzes the reduction of lipid hydroperoxides (LOOH) to non-reactive lipid alcohols, thereby suppressing ferroptosis.

Erastin and RAS-selective lethal small molecule 3 (RSL3) are well-established ferroptosis inducers (74). Erastin binds to SLC7A11 and inhibits its function, impairing cystine import and reducing GSH synthesis. This leads to the accumulation of lipid ROS and subsequent membrane damage, culminating in ferroptotic cell death (75). Additionally, erastin disrupts mitochondrial membranes by targeting voltage-dependent anion channels (VDACs), causing intracellular release of reactive species and further promoting cell death (76). In contrast, RSL3 directly covalently binds to GPX4, rendering it inactive and disrupting the redox balance. This results in elevated lipid peroxidation and induction of ferroptosis (77).

Transcription factors regulate SLC7A11 expression and thus modulate ferroptosis. The transcription factor NFE2L2 (Nrf2) positively regulates *SLC7A11* expression, whereas tumor suppressors such as TP53, BAP1, and BECN1 downregulate *SLC7A11*, increasing ferroptosis sensitivity (78–80). Therefore, targeting GSH metabolism represents a promising avenue for modulating ferroptosis in disease contexts.

#### 3.1.2 Relationship between lipid metabolism and ferroptosis

PUFAs, particularly those containing bis-allylic hydrogen atoms such as arachidonate (AA) and adrenate (AdA), are highly susceptible to oxidation by ROS, making them central to ferroptosis (81). Phosphatidylethanolamines (PEs) enriched in AA or AdA serve as critical substrates for lipid peroxidation during ferroptosis. Acyl-CoA synthetase long-chain family member 4 (ACSL4) catalyzes the conjugation of free AA or AdA with coenzyme A (CoA) to form AA-CoA or AdA-CoA. These derivatives are subsequently esterified into membrane PEs by lysophosphatidylcholine acyltransferase 3 (LPCAT3) (82). Thus, enhancing PUFA availability increases ferroptosis susceptibility, while inhibition of ACSL4 or LPCAT3 suppresses ferroptosis (83). The generation of CoA-conjugated PUFAs and their incorporation into phospholipids represent key steps in ferroptosis signaling, offering potential therapeutic targets for diseases associated with this mode of cell death.

Lipoxygenases (LOXs), iron-containing enzymes, play a direct role in inducing ferroptosis by catalyzing the peroxidation of membrane-bound PUFAs. In p53-mediated ferroptosis, p53 activates arachidonate 12-lipoxygenase (ALOX12), triggering ferroptosis independently of GPX4 activity (84). Targeting ALOX12—either pharmacologically or genetically—represents a novel strategy for preventing ferroptotic neuronal injury (85).

#### 3.1.3 Relationship between iron metabolism and ferroptosis

Iron exists in two major oxidation states: Fe²^+^ and Fe³^+^. Dietary Fe³^+^ is reduced to Fe²^+^ in the gastrointestinal tract before being absorbed by intestinal epithelial cells (86). Within cells, Fe²^+^ is exported via the iron transporter ferroportin 1 (FPN1) and oxidized to Fe³^+^ by multicopper oxidases. Fe³^+^ then binds to transferrin (TF) to form the TF-Fe³^+^ complex, which circulates and delivers iron to various tissues (87). Upon binding to transferrin receptor (TfR1) at the cell surface, TF-Fe³^+^ enters the cell via endocytosis. Intracellular STEAP3, a metalloreductase, reduces Fe³^+^ to Fe²^+^, which is then released into the labile iron pool (LIP) through the action of divalent metal transporter 1 (DMT1) (87). Under physiological conditions, the LIP maintains iron homeostasis. However, under pathological conditions, excessive Fe²^+^ drives the Haber–Weiss and Fenton reactions, producing high levels of ROS. These radicals initiate lipid peroxidation of PUFAs within membrane phospholipids, leading to membrane disruption and ferroptosis (88). Moreover, Fe²^+^ functions as a cofactor for multiple metabolic enzymes—including LOXs and PDH1—enhancing their activity and further stimulating ROS production (89). Therefore, components of iron metabolism represent potential therapeutic targets in ferroptosis regulation. The tumor suppressor OTUD1 interacts with and promotes deubiquitination of IREB2 (iron-responsive element-binding protein 2), stabilizing IREB2 and activating downstream TFRC gene expression. This enhances Fe²^+^ influx, elevates ROS levels, and increases cellular sensitivity to ferroptosis. Knockdown of IREB2 reduces ferroptosis susceptibility (90, 91). Furthermore, studies suggest that inhibition of transferrin (TF) activity can reduce Fenton reaction-driven ROS accumulation and lipid peroxidation, thereby attenuating ferroptosis (90). Deferoxamine (DFO), an iron chelator, prevents ferroptosis caused by iron overload, and limiting iron intake can slow liver injury induced by ferroptosis (92). Despite these findings, the precise mechanisms linking iron metabolism to ferroptosis remain incompletely understood and warrant further investigation.

#### 3.1.4 Redox stress-metabolic pathways regulating ferroptosis

Additional regulators of ferroptosis include coenzyme Q10 (CoQ10), NADPH, selenium, p53, NRF2/NFE2L2, and vitamin E. Among these, CoQ10, when reduced by ferroptosis suppressor protein 1 (FSP1), acts to prevent lipid oxidation and suppress ferroptosis (93). This suggests that FSP1 may serve as a promising therapeutic target in diseases involving ferroptosis. NADPH plays a crucial role in the regeneration of the GSH–GPX4 axis. Depletion of NADPH compromises antioxidant capacity, tipping the redox balance toward ferroptosis (94, 95). Selenium, an essential trace nutrient, contributes to GPX4 stability and enzymatic activity by cooperating with transcription factors TFAP2c and Sp1, exerting protective effects against ferroptosis in neurons (96). The NFE2L2 (Nrf2) pathway constitutes a key cellular defense against ferroptosis. NFE2L2 transactivates multiple genes involved in iron and GSH metabolism, as well as ROS detoxification, thereby limiting oxidative damage (67). As a major endogenous antioxidant, vitamin E protects cell membranes from lipid peroxidation. Studies show that vitamin E synergizes with GPX4 to suppress ferroptosis by inhibiting lipoxygenase (ALOX) activity and reducing lipid peroxide formation (97). Thus, vitamin E holds therapeutic promise as a ferroptosis-targeted intervention. Transcription factors p53 and NRF2 also play significant roles in ferroptosis. Although p53’s involvement in various forms of cell death has been studied for over two decades, its role in ferroptosis was only recently uncovered (98). p53 induces ferroptosis by repressing *SLC7A11* expression and blocking cystine uptake. Nutlin-3, an inhibitor of murine double minute 2 (MDM2), enhances p53 stability and preserves intracellular GSH levels via a p53-dependent mechanism, allowing cell survival under cystine-deprived conditions (98). Conversely, p53 can inhibit dipeptidyl peptidase-4 (DPP4), counteracting erastin-induced ferroptosis. Loss of p53 promotes DPP4–NADPH oxidase 1 (NOX1) interactions, forming a NOX1–DPP4 complex that mediates plasma membrane lipid peroxidation and triggers ferroptosis (78). NRF2 (also known as NFE2L2) is a central regulator of redox homeostasis. Through the p62–Keap1–NRF2 pathway, NRF2 upregulates the expression of genes involved in iron and ROS metabolism, such as *NQO1*, *HO1*, and *FTH1*, thereby suppressing ferroptosis (99). Evidence also indicates that *SLC7A11* is a transcriptional target of NRF2, suggesting that SLC7A11 may mediate the protective effects of NRF2. Further studies are required to validate this interaction (99).

### 3.2 Regulatory mechanisms of ferroptosis

**Figure 2:**
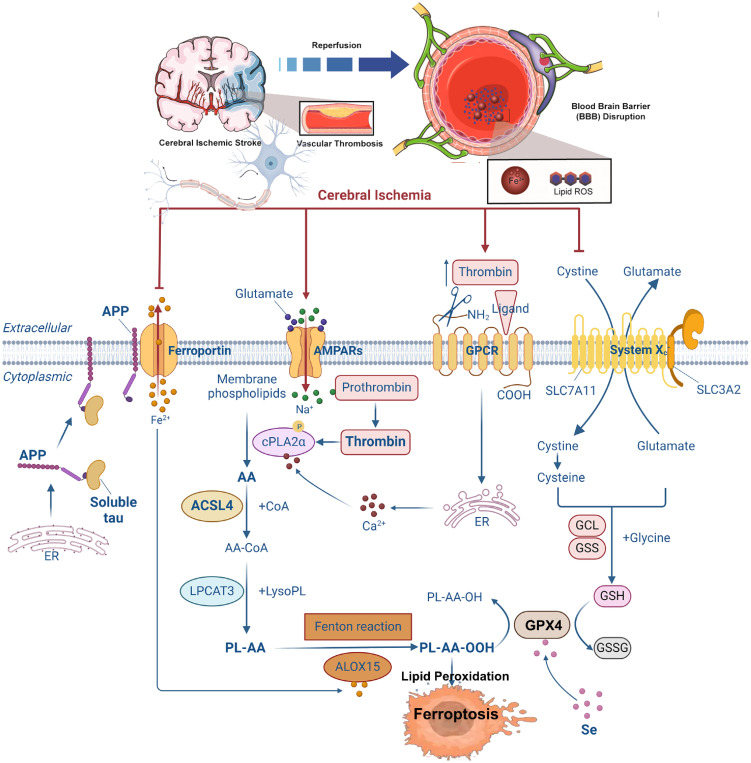
Pathways regulating ferroptosis. Different molecules regulate lipid peroxidation in multiple ways. BH4, tetrahydrobiopterin; GCH1, recombinant GTP cyclohydrolase 1; GPX4, glutathione peroxidase 4; DHFR, dihydrofolate reductase; FSP1: ferroptosis-suppressor protein-1.

As research progresses, scientists have uncovered a complex and finely tuned regulatory network governing ferroptosis, involving both inhibitory and activatory pathways, as illustrated in **Figure 2**.

#### 3.2.1 Inhibitory pathways of ferroptosis

The ferroptosis-inhibiting mechanisms include the classical GSH–GPX4 axis and several GPX4-independent pathways (100). 1) GSH–GPX4 antioxidant system in the cytosol and mitochondria: One of the most critical defenses against ferroptosis is the GSH–GPX4 system located in both the cytosol and mitochondria (101). On the cell membrane, the cystine/glutamate antiporter SLC7A11/SLC3A2—also known as system Xc^−^—transports extracellular cystine into the cell in a 1:1 exchange with intracellular glutamate release (101). Once inside the cell, cystine is reduced to L-cysteine, which is then used by glutamate-cysteine ligase (GCL) and glutathione synthetase (GSS) to synthesize GSH, with NADPH serving as a key reducing cofactor. Finally, GPX4 utilizes GSH to reduce lipid hydroperoxides into non-reactive lipid alcohols, thereby suppressing ferroptosis (102). 2) GPX4-independent ferroptosis suppression—FSP1–CoQ10 pathway: Myristoylated ferroptosis suppressor protein 1 (FSP1), also known as apoptosis-inducing factor mitochondrial 2 (AIFM2), resides on the plasma membrane and functions as a CoQ10 reductase. It plays a pivotal role in capturing lipophilic radicals and preventing lipid peroxide formation (103, 104). 3) GPX4-independent ferroptosis suppression—GCH1–BH4 pathway: GTP cyclohydrolase I (GCH1) and its metabolite tetrahydrobiopterin/dihydrobiopterin (BH4/BH2) protect cells from ferroptosis by selectively inhibiting the consumption of phospholipids containing two polyunsaturated acyl chains (105, 106). 4) GPX4-independent ferroptosis suppression—DHODH–CoQ10H_2_ pathway in the mitochondria: Dihydroorotate dehydrogenase (DHODH), a flavin-dependent enzyme localized to the mitochondrial inner membrane, catalyzes the fourth step of pyrimidine biosynthesis—converting dihydroorotate (DHO) to orotate (OA)—while transferring electrons to mitochondrial ubiquinone, reducing it to CoQ10H_2_, which acts as a radical-trapping antioxidant (107). Studies show that under conditions of low GPX4 expression, DHODH effectively suppresses mitochondrial lipid peroxidation and ferroptosis. However, when GPX4 levels are high, combining system Xc^−^ inhibitors such as sulfasalazine with DHODH inhibitors can synergistically induce ferroptosis (107).

#### 3.2.2 Activating pathways of ferroptosis

Lipid peroxidation serves as the final execution mechanism of ferroptosis. The synthesis of free fatty acids via acetyl-CoA carboxylase (ACAC) or their liberation through lipophagy leads to intracellular accumulation of free fatty acids, promoting ferroptosis (108). Long-chain acyl-CoA synthetase family member 4 (ACSL4) facilitates the incorporation of PUFAs, including arachidonate and adrenate, into cellular membranes (82). These PUFAs are subsequently esterified into phospholipids by lysophosphatidylcholine acyltransferase 3 (LPCAT3), generating PUFA-phospholipids (PUFA-PLs) that are highly susceptible to oxidation by lipoxygenases (ALOXs), ultimately leading to ferroptosis (109, 110). Iron availability is a defining feature of ferroptosis. Fe²^+^ drives the Fenton reaction, producing unstable hydroxyl radicals that initiate lipid oxidation of PUFAs, forming conjugated dienes and eventually yielding toxic lipid peroxidation products such as 4-hydroxynonenal (4-HNE) and MDA. These molecules increase membrane fragility, impair cellular function, and promote ferroptotic death (91, 111). Several key genes in iron metabolism that regulate ferroptosis were identified, including transferrin and SLC39A14 iron transporters (112), the heavy chain of ferritin (FTH1) (113, 114), as well as TFR1 (115), FPN1 (116, 117), PCBP1 (118), and NCOA4 (119)—all reported to modulate ferroptosis through iron homeostasis. These findings highlight the central role of iron metabolism in ferroptosis regulation and underscore the need for further mechanistic exploration.

### 3.3 Pharmacological modulation of ferroptosis

Both inducers and inhibitors of ferroptosis have emerged as potential therapeutic agents, targeting different nodes along the ferroptotic pathway.

#### 3.3.1 Ferroptosis inducers

Small molecules targeting iron metabolism: Erastin was first identified in 2003 as a genetically selective antitumor agent that induces ferroptosis primarily through interaction with VDACs (76, 120). Another compound, RSL5, binds VDAC3 and similarly triggers ferroptosis. VDACs can be allosterically blocked by 4,4′-diisothiocyanatostilbene-2,2′-disulfonic acid (DIDS), a small-molecule inhibitor of mitochondrial anion channels. Temozolomide (TMZ), an anticancer drug used in glioblastoma treatment, enhances DMT1 activity and promotes ferroptosis. A recently discovered ferroptosis inducer, MMRi62, induces degradation of the ferritin heavy chain (120).

Small molecules targeting lipid metabolism: The antitumor agent sorafenib, in the presence of ACSL4, activates ferroptosis by directly affecting lipid ROS generation pathways (121). Similarly, tert-butyl hydroperoxide (t-BuOOH) increases lipid ROS production, inducing oxidative stress, mitochondrial depolarization, and DNA damage. t-BuOOH exerts its effect through cardiolipin oxidation, and this process can be reversed by cardiolipin oxidation inhibitors such as XJB-5–131 and JP4–039 (121).

Small molecules targeting the GSH/GPX4 axis: RSL3 and RSL5, although functionally distinct, were among the earliest identified GPX4-targeted ferroptosis inducers. RSL3 covalently binds to the active site of GPX4, inactivating its enzymatic function (77). Other compounds, such as ML162, DPI7, and DPI10, exhibit similar effects. Additionally, FIN56 accelerates GPX4 degradation, promoting ferroptosis. Besides erastin and its analogs, several FDA-approved drugs have been shown to inhibit the system Xc^−^. For example, sulfasalazine (SAS) suppresses system Xc^−^ and exhibits antitumor properties. Sorafenib indirectly inhibits system Xc^−^ by blocking upstream regulators and also reduces GPX4 activity. iv) Small molecules targeting the FSP1/CoQ pathway: In addition to erastin, FIN56 is another multimodal inducer of ferroptosis. FIN56 not only promotes GPX4 degradation but also binds squalene synthase (SQS) and depletes CoQ, thereby triggering ferroptosis (122). NDP4928, a specific FSP1 inhibitor, sensitizes cells to GSH depletion by binding and inhibiting FSP1. Statins, formulated as therapeutic nanoparticles, block HMG-CoA reductase (HMGCR) in the mevalonate pathway, thereby reducing CoQ levels and promoting ferroptosis (122).

#### 3.3.2 Ferroptosis inhibitors

Iron-lowering agents: Ciclopirox olamine (CPX) is an iron chelator with broad-spectrum antifungal and antibacterial activity. Beyond its chelating capacity, CPX-O has been shown to induce ferritin degradation in polycystic kidney disease mouse models (123, 124). DFO is a widely used iron chelator that has demonstrated efficacy in traumatic spinal cord injury. Additional iron chelators such as deferiprone (DFP) and deferasirox (DFX) have also been reported to inhibit ferroptosis (124).

Lipid peroxidation inhibitors: Ferrostatin-1 (Fer-1) suppresses lipid peroxidation and rescues p53-driven ferroptosis (125). α-Tocopherol (vitamin E) and structurally related analogs of Fer-1 share similar protective functions, though with variable efficacy and stability. Recent studies have identified vitamin K hydroquinone (VKH2), the fully reduced form of vitamin K, as a potent inhibitor of lipid oxidation and ferroptosis. iii) Modulators of the GSH/GPX4 axis: Small molecules such as β-mercaptoethanol (β-ME) prevent ferroptosis by stabilizing redox balance (126). Selenium (Se) supplementation enhances GPX4 expression, offering protection against ferroptotic insults. In short, the development of ferroptosis inducers and inhibitors is essential for dissecting the molecular basis of this unique form of regulated cell death. As new targets and pathways continue to emerge, they provide a foundation for developing novel therapeutics across a spectrum of diseases where ferroptosis plays a pathogenic role, including cancer, neurodegenerative disorders, and ischemia–reperfusion injury.

## 4 Ferroptosis in neurodegenerative diseases: mechanistic insights

Ferroptosis is a regulated form of cell death driven by iron-dependent lipid peroxidation, and mounting evidence suggests its involvement in the pathogenesis of neurodegenerative diseases, including ischemic stroke, PD, AD, and HD (127). The mechanisms underlying ferroptosis—such as iron overload, lipid metabolism dysregulation, and antioxidant defense failure—are increasingly recognized as key contributors to neuronal loss and functional deterioration in these disorders. This section will discuss classical neurodegenerative diseases (such as AD, PD, HD, and ALS), as well as other acute and chronic neurological disorders known to involve ferroptosis, including ischemic stroke, traumatic brain injury (TBI), and Friedreich’s ataxia (FRDA).

### 4.1 Ferroptosis in cerebral ischemia

**Figure 3:**
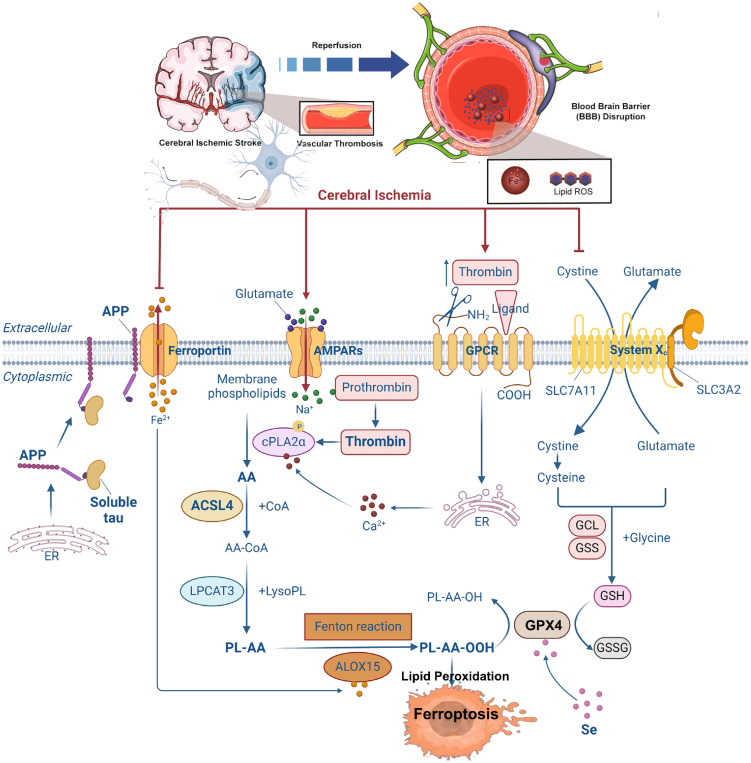
Summary of the mechanisms of ferroptosis in cerebral ischemia. Following cerebral ischemia, energy depletion impairs the clearance of excitatory neurotransmitters from the synaptic cleft, leading to the accumulation of glutamate and other excitatory signals. This excess glutamate activates AMPA receptors, promoting Na^+^ influx into neurons. Elevated intracellular Na^+^ levels trigger the conversion of prothrombin into active thrombin, which in turn induces phosphorylation and activation of calcium-dependent cPLA2α. Additionally, thrombin from the bloodstream may enter the brain through a compromised blood–brain barrier, further activating cPLA2α by increasing cytosolic Ca²^+^ concentrations. Activated cPLA2α hydrolyzes membrane phospholipids at the sn-2 position to release AA. AA is then converted to AA-CoA by ACSL4 and subsequently incorporated into membrane phospholipids via LPCAT3, forming PL-AA. Under the catalytic action of ALOX15 and the Fenton reaction, PL-AA undergoes peroxidation, generating bioactive lipid peroxides that contribute to ferroptosis. GPX4 normally reduces LOOH to non-toxic LOH. However, elevated extracellular glutamate inhibits cystine uptake, limiting GSH synthesis. This depletion of GSH inactivates GPX4, leading to the accumulation of toxic lipid peroxides and promoting ferroptosis. Moreover, ischemia-induced reduction in soluble tau protein impairs the transport of APP to the neuronal membrane. This disruption inhibits the interaction between APP and Fpn, blocking Fe²^+^ export and resulting in intracellular Fe²^+^ accumulation and iron-dependent cell death. cPLA2α, cytosolic phospholipase A2α; AA, arachidonic acid; ACSL4, acyl-CoA synthetase long-chain family member 4; LPCAT3, lysophosphatidylcholine acyltransferase 3; PL-AA, AA-containing phospholipids; ALOX15, arachidonate 15-lipoxygenase; GPX4, glutathione peroxidase 4; LOOH, lipid peroxides; LOH, lipid alcohols; GSH, glutathione; APP, amyloid precursor protein; Fpn, ferroportin.

Stroke, also known as cerebral ischemia, is a leading cause of global mortality and disability. According to the GBD 2021 study published in *The Lancet Neurology*, 11.9 million individuals experienced their first stroke in 2021, resulting in 7.25 million deaths and 160 million disability-adjusted life years (DALYs) (127). Stroke ranks as the third leading cause of death and the fourth leading cause of disability worldwide (127). Ischemic stroke occurs when blood flow to specific brain regions is restricted due to occlusion of the middle cerebral artery, vertebral/basilar artery, or internal carotid artery (128). This deprivation of oxygen and nutrients activates an ischemic cascade, leading to oxidative stress, mitochondrial dysfunction, and ultimately neuronal death (129). Ferroptosis has emerged as a major mechanism of neuronal death in this context, with studies showing that inhibition of ferroptosis can prevent ischemia/reperfusion injury (130–151). In the middle cerebral artery occlusion (MCAO) model, mice treated with ferroptosis inhibitors exhibit reduced brain damage, indicating that ferroptosis contributes significantly to post-ischemic neuronal death. Notably, tau knockout mice are resistant to ferroptotic cell death following reperfusion, suggesting that the tau–iron interaction serves as a pleiotropic modulator of ferroptosis and ischemic stroke progression (130). Current understanding attributes ferroptosis in ischemic stroke to several factors, including intracellular iron accumulation, lipoxygenase activation, GSH depletion, GPX4 inactivation, and system Xc^−^ disruption (130) (**Figure 3**).

#### 4.1.1 Role of iron metabolism in ischemic stroke

Prior to the formal identification of ferroptosis, studies had already linked iron accumulation to neuronal damage in both clinical settings and animal models of cerebral ischemia (35, 36, 131). Excessive iron exacerbates mitochondrial oxidative damage and enlarges infarct size (19, 37, 132). In children, increased iron deposition is observed in the basal ganglia, thalamus, and periventricular white matter after severe hypoxic–ischemic injury (25, 130), reinforcing the role of disrupted iron homeostasis in ferroptosis and stroke pathology. Iron overload following ischemia may be mediated through two primary pathways: IL-6–JAK/STAT3–hepcidin axis: Ischemia-induced IL-6 activates the JAK/STAT3 signaling pathway, which enhances hepcidin expression. Elevated hepcidin downregulates FPN1, impairing iron export and promoting cellular iron retention and subsequent ferroptosis (133). However, the precise mechanisms driving IL-6 upregulation during ischemia remain incompletely understood. HIF-1α–TFR1 axis: Hypoxia-inducible factor 1α (HIF-1α) is upregulated during ischemia, enhancing transferrin receptor 1 (TFR1) expression. Since TFR1 mediates the main route of iron entry into neurons via the Tf-TFR1 complex, HIF-1α-dependent TFR1 induction leads to iron influx and overload within neurons (134). These findings highlight the central role of iron dyshomeostasis in the pathophysiology of ischemic stroke and suggest that targeting iron metabolism could represent a viable therapeutic strategy.

#### 4.1.2 Role of lipid metabolism in ischemic stroke

Studies indicate that ACSL4 is overexpressed after cerebral ischemia and contributes to reperfusion injury (135). miR-347 has been implicated in ACSL4 regulation, with elevated miR-347 levels observed in stroke patients correlating with increased ACSL4 expression (135). Conversely, other studies report decreased ACSL4 levels in the hippocampus of MCAO rats after reperfusion, coinciding with elevated thrombin activity. This downregulation appears to be an early event independent of neuronal death, potentially mediated by thrombin-induced ACSL4 degradation and ferroptosis promotion (136). Downregulation of ACSL4 may thus serve as a protective mechanism against ferroptosis during reperfusion. Further research confirms that ACSL4 suppression in early ischemic stages is time-dependent and likely driven by increased HIF-1α, which binds to the ACSL4 promoter and suppresses its expression (137). Lipoxygenases (LOXs), particularly 12/15-LOX, play a pivotal role in ferroptosis-mediated injury. These enzymes not only induce neuronal damage but also promote nuclear translocation of apoptosis-inducing factor (AIF) (138, 139). Inhibition of 12/15-LOX reduces neuronal and vascular injury following stroke (140). 5-LOX overexpression exacerbates inflammation and neuronal damage after ischemia/reperfusion (I/R). Conversely, miR-193b-3p, which targets 5-LOX, exerts neuroprotective effects in I/R injury, identifying it as a potential therapeutic target (141, 142).

#### 4.1.3 Role of amino acid metabolism in ischemic stroke

Selenium activates transcription factors such as Sp1 and TFAP2c, thereby enhancing GPX4 expression and inhibiting ferroptosis (143, 144). Experimental models show that GPX4 overexpression alleviates cerebral injury after stroke, whereas GPX4 deletion exacerbates infarction (145). The cystine/glutamate antiporter system Xc^−^, composed of SLC7A11 and SLC3A2, supports GSH synthesis and maintains redox homeostasis (146). Under normal conditions, this system facilitates cystine uptake and glutamate efflux in a non-ATP-dependent manner. During stroke, however, elevated extracellular glutamate causes excitotoxicity, blocking system Xc^−^ function and reducing GSH synthesis, thereby triggering ferroptosis (147). Impaired system Xc^−^ activity has been linked to xCT subunit inactivation (148), which sustains glutamate excitotoxicity in rat models of cerebral ischemia (149). At high concentrations, system Xc^−^ may paradoxically enhance glutamate toxicity and ferroptosis. The exact contribution of system Xc^−^ to ferroptosis in human stroke remains to be fully elucidated.

#### 4.1.4 Therapeutic targeting of ferroptosis in ischemic stroke

The primary goal in treating ischemic stroke is rapid reperfusion, a prerequisite for effective neuroprotection. Currently, tissue plasminogen activator (tPA) is the only FDA-approved thrombolytic agent for acute ischemic stroke (149). Despite ongoing efforts, most neuroprotectants have failed to demonstrate significant benefits in clinical trials, underscoring the need for novel mechanistic approaches. Iron chelators offer a promising avenue for intervention. DFO, an FDA-approved iron chelator, effectively reduces hydroxyl radical generation and attenuates brain injury in experimental stroke models (150, 151). Although DFO shows preclinical efficacy, its clinical translation remains limited, necessitating further evaluation of safety and effectiveness in stroke patients. Small-molecule ferroptosis inhibitors such as Fer-1 and liproxstatin-1 (Lip-1) inhibit ferroptosis induced by system Xc^−^ blockade or GPX4 loss (130). Delayed administration of Lip-1 (6 h post-reperfusion) still provides neuroprotection by preventing sustained neuronal damage (25, 130). Intranasal delivery of Fer-1 improves neurological deficits and reduces infarct volume 24 h after MCAO (25, 130). Recent advances have led to the development of new ferroptosis inhibitors, including phenothiazine derivatives, which exhibit potent neuroprotective effects in ischemic stroke models (152). These compounds act similarly to classical antioxidants by capturing reactive radicals, offering a novel approach to lipid peroxidation suppression.

Upregulating GPX4 represents another promising therapeutic strategy. Cysteamine, a cystamine derivative, elevates GSH levels in cortical neurons and protects against ferroptosis (153). In animal models, cystamine reduces infarct volume and promotes recovery (154). Selenium-based interventions also activate protective gene expression programs that enhance GPX4 and counteract excitotoxicity and ER stress-induced neuronal death (155). Additionally, cystamine and ornithine-threonine-cysteine (OTC), a cysteine prodrug, protect neurons from oxygen–glucose deprivation (OGD)-induced death by increasing GSH levels in the penumbra after reperfusion, demonstrating neuroprotection (156). Moreover, lipid ROS sensors such as Liperfluo provide real-time assessment of lipid peroxidation and ferroptosis, enabling dynamic monitoring of redox status in ischemic brain tissues (16). These tools support the development of lipid-targeted therapies for stroke. Selective 5-LOX inhibitors like zileuton reduce leukotriene production and dampen inflammatory responses during reperfusion (157). In transient global cerebral ischemia models, zileuton lowers inflammatory cytokines and chemokines (158), and in MCAO rats, it improves neurological outcomes and reduces infarct volume compared to controls (159). While zileuton demonstrates neuroprotective properties in animal models, direct evidence linking its effects to ferroptosis inhibition remains lacking. In contrast, 12/15-LOX has been directly implicated in ferroptosis-driven neuronal injury. siRNA-mediated knockdown of 12/15-LOX confers resistance to ferroptosis *in vitro* and protects neurons in ischemic mouse models (160). Pharmacological inhibition of 12/15-LOX using agents such as ML351, LOXBlock-1, and BW-B70C significantly reduces infarct volume and mitigates poststroke brain damage (161–163). These findings position 12/15-LOX as a critical mediator of ferroptosis in ischemic stroke and a promising drug target for neuroprotection.

#### 4.1.5 Natural compounds and traditional Chinese medicine targeting ferroptosis in ischemic stroke

Traditional Chinese medicine (TCM) categorizes ischemic stroke under “Zhong Feng” (中风), with acupuncture, herbal extracts, and compound formulations playing integral roles in treatment. Accumulating evidence suggests that TCM interventions exert neuroprotective effects through modulation of ferroptosis. Electroacupuncture (EA), an advanced form of acupuncture incorporating electrical stimulation, has shown beneficial effects in ischemic stroke models. EA reduces OGD/R-induced ferroptosis in hippocampal neurons; decreases ACSL4, Tf, and GSK-3β expression; and increases GPX4, Wnt1, and β-catenin levels, implicating the Wnt/β-catenin pathway in EA-mediated neuroprotection (164). In MCAO rats, EA applied at specific acupoints such as Zusanli (ST36) and Quchi (LI11) reduces iron overload and oxidative stress, ameliorates mitochondrial injury, and limits ferroptosis (165, 166). Overall, EA regulates ferroptosis by modulating iron-related proteins and redox homeostasis (167). Network pharmacology approaches have revealed multiple active ingredients and signaling pathways in TCM that influence ferroptosis. Ou et al. identified six TCM compounds acting on multiple ferroptosis-related targets, and 7 out of 15 predicted ferroptosis regulators sensitive to TCM modulation (168). This highlights the potential of TCM in targeting ferroptosis for stroke therapy. For example, rehmannioside A, a glycoside isolated from *Rehmannia glutinosa*, improves cognitive deficits and neurological function in MCAO rats. Its neuroprotective effects are associated with activation of the PI3K/AKT/NRF2 and SCL7A11/GPX4 signaling pathways, indicating a multitarget mechanism of action (169).

Baicalin, a flavone derived from *Scutellaria baicalensis*, suppresses ferroptosis markers such as iron levels, lipid peroxidation products, and morphological changes in tMCAO mice. It also inhibits RSL3-induced ferroptosis in HT22 cells and reverses I/R injury through the GPX4/ACSL4/ACSL3 axis and PINK1-Parkin-mediated mitochondrial autophagy regulation (170, 171). Similarly, galangin, a flavonoid extracted from *Alpinia officinarum*, activates the SCL7A1/GPX4 axis and suppresses ferroptosis in gerbil hippocampal neurons after I/R injury (172). Astragaloside IV, a saponin from *Astragalus membranaceus*, reduces infarct volume and neuronal damage in MCAO rats, potentially through modulation of transmembrane iron transport and ferroptosis pathways (173). However, the specific bioactive components responsible for this effect remain to be characterized. Danhong injection (DHI), a standardized formulation containing *Salvia miltiorrhiza* and *Carthamus tinctorius*, is widely used in cardiovascular and cerebrovascular disease management (174). In MCAO mice, DHI treatment significantly reduces infarct size, mitochondrial necrosis, and iron accumulation, highlighting its ability to suppress ferroptosis and improve stroke outcomes (175). Another TCM formula, Compound Tongluo Decoction (CTLD), reduces infarct volume, alleviates endoplasmic reticulum (ER) stress, and stimulates angiogenesis. Mechanistically, CTLD suppresses ferroptosis via activation of the Sonic Hedgehog (Shh) pathway (176). Our group previously demonstrated that Naotai Formula (NTF) enhances the HSF1/HSPB1 pathway and regulates brain iron homeostasis by suppressing TFR1/dMT1 and activating SCL7A11/GPX4, thereby protecting against ferroptosis in MCAO rats (177, 178). Although current data support the therapeutic potential of TCM in targeting ferroptosis after stroke, the multicomponent and multitarget nature of these interventions necessitates deeper mechanistic exploration to optimize clinical translation.

Ferroptosis is a multifactorial process involving iron metabolism, lipid peroxidation, and antioxidant defense systems. In ischemic stroke, ferroptosis contributes to neuronal death and brain injury through mechanisms involving iron overload, LOX activation, and system Xc^−^ dysfunction. Emerging evidence indicates that targeting ferroptosis—either through small-molecule inhibitors, natural compounds, or TCM formulations—can mitigate brain damage and improve functional outcomes. Despite these promising findings, several challenges remain. First, the molecular triggers of ferroptosis in stroke are not fully understood, and specific biomarkers distinguishing ferroptosis from other forms of programmed cell death are currently lacking. Second, most evidence is derived from preclinical models, and large-scale clinical validation is required. Third, while TCM offers multitarget neuroprotection, its mechanisms and active components remain poorly defined. Future research should focus on identifying key regulatory nodes in ferroptosis, validating novel therapeutics, and dissecting the molecular basis of TCM interventions. By integrating these approaches, we can develop more effective and personalized strategies for managing ischemic stroke and related neurodegenerative conditions.

In summary, although current studies on ferroptosis in cerebral ischemia have identified key biological processes such as lipid peroxidation and iron metabolism imbalance, two central challenges remain unresolved. First, the specificity and clinical utility of ferroptosis biomarkers in human patients have yet to be established. Biomarkers validated in animal models—such as serum GPX4 activity and cerebrospinal fluid 4-HNE levels—show marked individual variability among ischemic stroke patients and are strongly influenced by reperfusion timing and comorbidities such as diabetes. At present, no multicenter cohort studies have validated biomarkers with both diagnostic and prognostic value, nor has the issue of *in vivo* molecular imaging sensitivity been overcome, particularly for PET probes targeting transferrin receptors. Second, the interaction between ferroptosis and other cell death pathways (such as apoptosis and pyroptosis) remains poorly defined across brain regions. In the ischemic penumbra, neurons predominantly undergo ferroptosis, while astrocytes in the ischemic core are more prone to pyroptosis. However, the signaling intersections between these pathways—for example, whether the NLRP3 inflammasome regulates ferritin heavy chain degradation—remain controversial. Existing reviews tend to list these interactions without critically addressing the gap in understanding of cell-type-specific interaction axes.

In contrast, the innovation of this review lies in three key aspects. 1) By integrating 2025 single-cell sequencing and proteomic datasets, it incorporates vascular endothelial cells for the first time and constructs a three-dimensional map of ferroptosis heterogeneity across neurons, glia, and endothelium. This allows us to propose a novel “stage–cell type–regulator” network model that addresses the limitation of previous reviews which overlooked cellular heterogeneity. 2) To overcome the biomarker bottleneck, we introduce a dual-dimensional diagnostic strategy that combines exosomal proteins and miRNAs, thereby enhancing subtype specificity and improving diagnostic synergy. 3) By linking iron-mediated ferroptosis with necroptosis-like apoptotic features, we propose a “nanocarrier-based dual-target inhibitor” design tailored to specific reperfusion stages. This interdisciplinary approach provides a theoretical framework for precision intervention and offers a more targeted foundation for clinical translation.

### 4.2 Ferroptosis in intracerebral hemorrhage

Intracerebral hemorrhage (ICH), a form of intracranial bleeding, is a life-threatening cerebrovascular disorder associated with high mortality and long-term disability worldwide (179, 180). ICH is characterized by sudden onset, rapid progression, and multiple complications, contributing to its substantial clinical burden. Current management primarily relies on surgical intervention to alleviate primary injury; however, no effective therapies exist for secondary brain injury after ICH (SBI-ICH) (180). The pathophysiology of ICH involves both primary mechanical damage from hematoma expansion and secondary biochemical and biomechanical alterations that exacerbate neuronal injury (181). In particular, the degradation of hemoglobin from lysed red blood cells releases toxic substances—such as iron—that contribute to oxidative stress, neuroinflammation, and blood–brain barrier disruption (182). A 2017 study was the first to demonstrate activation of ferroptotic pathways following ICH (183), and subsequent studies have confirmed that ferroptosis contributes to neuronal damage after hemorrhage via mechanisms involving neurotoxin release and oxidative stress (184, 185). Following ICH, iron released from hemoglobin triggers excessive ROS production, leading to oxidative stress and secondary brain injury. Preclinical and clinical evidence indicates that toxins derived from hematomas can induce neuronal death through ferroptosis. Specifically, during ICH, erythrocyte lysis leads to hemoglobin release, which is subsequently phagocytized by activated microglia and macrophages. These cells reduce Fe³^+^ to Fe²^+^, which is then exported via FPN1 and transported into neurons via the transferrin–transferrin receptor system. Within neurons, excess Fe²^+^ undergoes Fenton or Haber–Weiss reactions, generating hydroxyl radicals that attack DNA, proteins, and lipid membranes, thereby inducing ROS and lipid peroxidation (183). Therefore, targeting hemoglobin- and iron-induced toxicity may represent a novel therapeutic strategy for ICH.

**Figure 4:**
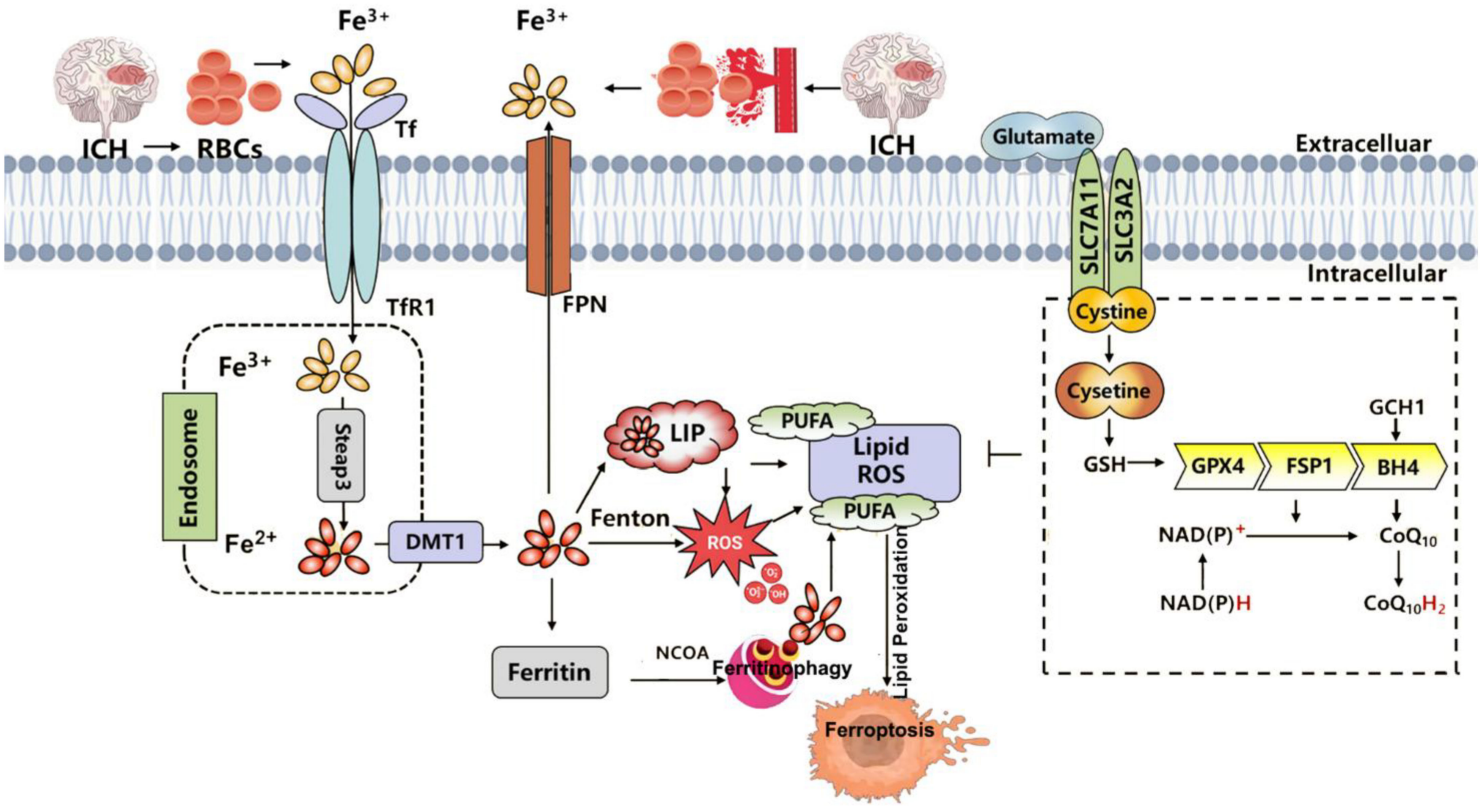
Summary of the mechanisms of ferroptosis in intracerebral hemorrhage (ICH). ICH leads to the extravasation of RBCs into brain tissue. Upon lysis, RBCs release hemoglobin, which is degraded into heme, releasing iron into the local microenvironment. Extracellular Fe³^+^ binds to Tf and is internalized through TfR1-mediated endocytosis. Within endosomes, Fe³^+^ is reduced to Fe²^+^ by Steap3 and transported into the cytoplasm via DMT1. Once in the cytosol, Fe²^+^ enters the LIP, where it may be stored in ferritin, released through ferritinophagy, or exported by FPN1. Excess Fe²^+^ catalyzes the Fenton reaction, generating ROS that drive lipid peroxidation of PUFAs, ultimately triggering ferroptosis and neuronal injury. Three major ferroptosis-suppressing pathways counteract this process: the system Xc^−^–GSH–GPX4 axis, which supports detoxification of lipid peroxides; the FSP1–CoQ10–NAD(P)H pathway, which prevents membrane lipid damage; and the GCH1–BH4 axis, which enhances CoQ10 synthesis and ROS clearance to alleviate oxidative stress. RBC, red blood cell; Tf, transferrin; TfR1, transferrin receptor 1; Steap3, six-transmembrane epithelial antigen of the prostate 3; DMT1, divalent metal transporter 1; LIP, labile iron pool; FPN1, ferroportin 1; ROS, reactive oxygen species; PUFA, polyunsaturated fatty acid; GPX4, glutathione peroxidase 4; GSH, glutathione; FSP1, ferroptosis suppressor protein 1; CoQ10, coenzyme Q10; NAD(P)H, nicotinamide adenine dinucleotide (phosphate): reduced form; GCH1, GTP cyclohydrolase 1; BH4, tetrahydrobiopterin.

In organotypic hippocampal slice cultures, the specific ferroptosis inhibitor Fer-1 prevents neuronal death and reduces hemoglobin-induced iron deposition. Mice treated with Fer-1 after ICH show significant neuroprotection and improved neurological function (183). Moreover, Fer-1 attenuates lipid ROS accumulation and suppresses the upregulation of PTGS2 (which encodes COX-2)—a marker of ferroptosis—supporting the involvement of ferroptosis in post-hemorrhagic neuronal loss (186). Iron-dependent cell death occurs not only at the core of the hematoma but also in remote brain regions, suggesting widespread ferroptosis after ICH (186). In this context, FPN1, the only known mammalian iron exporter, is significantly upregulated in aged ICH patients and mouse models. Genetic deletion of *Fpn1* worsens ICH outcomes, whereas viral overexpression of *Fpn1* improves recovery, indicating that enhancing iron efflux can mitigate ferroptosis after hemorrhage (117). Oxidized low-density lipoprotein receptor 1 (OLR1), also known as LOX-1, is upregulated in ICH hematomas. Silencing OLR1 in rat models reduces brain edema, neuronal loss, inflammation, and oxidative stress. This protective effect is mediated through upregulation of GPX4 and FTH1, and downregulation of COX-2, highlighting OLR1 as a key regulator of ferroptosis in ICH (187). Sphingosine kinase 1 (Sphk1) is another critical mediator of ferroptosis in ICH. Its expression is elevated after hemorrhage, and it functions in sphingolipid metabolism to promote ferroptosis (188). Inhibition of Sphk1 in both *in vivo* and *in vitro* ICH models attenuates secondary brain injury and neuronal death, reinforcing the role of Sphk1 in ferroptosis-driven ICH pathology. These findings suggest that targeting Sphk1 could offer a promising approach for preventing and treating ICH-related neurodegeneration. Collectively, these data support the hypothesis that ferroptosis is a major contributor to neuronal injury after ICH, potentially through modulation of ferroptosis-associated gene expression (**Figure 4**).

#### 4.2.1 Mechanisms of neuronal ferroptosis after intracerebral hemorrhage

Due to the presence of the blood–brain barrier (BBB), iron entry into the brain is tightly regulated. Under normal conditions, Fe³^+^ circulates bound to transferrin and enters neurons via TfR1-mediated endocytosis (189). Upon ICH, this balance is disrupted: increased extracellular hemoglobin enhances Fe²^+^ release, overwhelming cellular antioxidant defenses and triggering ferroptosis. Within the brain parenchyma, some Fe²^+^ is stored in the labile iron pool, while excess iron is exported via FPN1. However, in the setting of ICF, the brain’s limited antioxidant capacity makes it highly susceptible to oxidative damage (190, 191). Post-ICH, activated microglia phagocytize hemoglobin and release chemokines that recruit blood-derived macrophages to the hemorrhagic site. These cells metabolize hemoglobin into free iron, which is then transported into neurons via TfR1. The resulting Fenton reaction generates hydroxyl radicals, promoting extensive lipid peroxidation and damaging macromolecules such as proteins, lipids, and DNA, ultimately leading to neuronal death (192).

To counteract this, the body activates antioxidant systems such as the GPX4–GSH axis. However, during ICH, extracellular glutamate levels rise, inhibiting system Xc^−^ activity, reducing GSH synthesis, and impairing GPX4 function (193). Consequently, redox homeostasis is lost, lipid peroxidation cascades are initiated, and ferroptosis ensues.

#### 4.2.2 Ferroptosis exacerbates secondary brain injury post-ICH

Secondary brain injury after ICH results from complex interplay between brain edema, neuroinflammation, oxidative stress, and programmed cell death including autophagy, apoptosis, and ferroptosis (193). Ferroptosis intensifies these processes, worsening overall outcome. Hemoglobin and iron released from hematomas activate microglia, leading to elevated inflammatory cytokines and mediators (193). Fe²^+^ further drives ROS generation via Fenton chemistry, initiating lipid peroxidation and disrupting redox balance. Accumulated hydroxyl radicals and lipid peroxides accelerate the oxidation of biomolecules, amplifying oxidative stress and neuronal damage (157). Hydroxyl radicals generated via Fenton reactions directly increase cerebral edema by impairing ion homeostasis. ROS inhibit Na^+^-K^+^ ATPase and Ca²^+^ pumps, leading to intracellular ionic dysregulation, acidosis, and cytotoxic edema (194). Iron overload not only promotes lipid peroxidation but also induces apoptosis and autophagy, further aggravating secondary brain injury (195). These findings underscore that ferroptosis plays a central role in exacerbating ICH-induced brain damage and that interventions targeting this pathway may provide neuroprotection.

#### 4.2.3 Nrf2 activation suppresses ferroptosis and mitigates secondary brain injury after ICH

Activation of nuclear factor (erythroid-derived 2)-like 2 (Nrf2) has emerged as a promising strategy for limiting ferroptosis and improving functional outcomes after ICH. Withaferin A (WFA), a natural compound with neuroprotective properties, significantly reduces MDA levels and enhances antioxidant enzymes such as superoxide dismutase (SOD) and GPX in a murine model of striatal ICH (196). WFA activates the Nrf2/HO-1 pathway, promoting nuclear translocation of Nrf2 and increasing HO-1 expression. Knockdown of Nrf2 reverses these effects, confirming that WFA exerts neuroprotection via Nrf2-dependent suppression of ferroptosis. Furthermore, combining WFA with Fer-1 reduces hemin-induced neuronal damage in SH-SY5Y cells, reinforcing the therapeutic potential of dual ferroptosis inhibition (196). Peroxisome proliferator-activated receptor γ (PPARγ) synergizes with Nrf2 to enhance antioxidant gene expression and suppress ferroptosis. Administration of pioglitazone (PDZ), a PPARγ agonist, activates Nrf2 and GPX4 in primary rat hippocampal neurons exposed to hemin. In an autologous blood injection model of ICH, PDZ improves neurological recovery, an effect blocked by the Nrf2 inhibitor ML385, demonstrating that PPARγ/Nrf2/GPX4 signaling underlies its protective mechanism (197). Crocin, a carotenoid derived from saffron, alleviates brain edema and neurological deficits in a mouse model of ICH. Crocin increases SOD and GPX activity; elevates GPX4, FTH1, and SLC7A11 expression; and suppresses lipid peroxidation and iron accumulation, supporting its anti-ferroptotic function (198). Further mechanistic studies indicate that crocin exerts these effects through Nrf2 activation, offering a multitargeted approach to ameliorate secondary brain injury (198). (−)-Epicatechin (EC), a flavanol found in green tea and cocoa, reduces lesion volume and perihematomal neuronal death in experimental ICH. EC activates Nrf2, decreases HO-1 expression, and limits iron deposition, thereby suppressing ferroptosis and related gene expression (199). These findings reinforce the importance of Nrf2 in mitigating ferroptosis and improving stroke outcomes.

Collectively, these studies highlight that pharmacological activation of Nrf2 can reduce ferroptosis and improve neurological deficits in preclinical models of ICH.

#### 4.2.4 Therapeutic potential of ferroptosis inhibitors in ICH

Ferroptosis is closely linked to iron overload, impaired antioxidant defense, and lipid peroxidation in ICH, all of which contribute to inflammation and neuronal damage. Targeting these pathways offers new therapeutic opportunities for reducing secondary brain injury.

1. Iron chelators: Fer-1, a selective ferroptosis inhibitor, reduces ROS and lipid peroxidation, preserves neuronal viability, and improves neurological function in both *in vitro* and *in vivo* models (200). Iron chelators such as DFO, DFP, VK28, and clioquinol bind free Fe²^+^, forming stable complexes that limit iron toxicity. These agents inhibit microglial activation, reduce hemolysis-induced iron overload and oxidative stress, and protect neurons from ferroptosis (201, 202). Clioquinol also enhances Fpn1 expression, facilitating iron efflux and offering an additional route of ferroptosis inhibition (203). Minocycline, a tetracycline antibiotic, shows iron-chelating properties and protects aging female rats from ICH-induced brain injury, although its exact mechanism remains unclear (204). The lipophilic iron chelator pyridoxal isonicotinoyl hydrazone (PIH) reduces total iron, ROS, and lipid peroxidation in perihematomal tissue; increases GPX4 expression; shifts microglia toward the anti-inflammatory M2 phenotype; and suppresses pro-inflammatory cytokines, thereby exerting broad neuroprotection (205).
2. Lipid peroxidation inhibitors: Long non-coding RNA H19 promotes ferroptosis in brain microvascular endothelial cells by regulating the miR-106b-5p/ACSL4 axis. Knockdown of H19 increases SLC7A11 and GPX4 mRNA levels and downregulates TFR1, effectively blocking ferroptosis (206). Paeonol, a natural product isolated from *Paeonia suffruticosa*, inhibits ferroptosis via the HOTAIR/UPF1/ACSL4 axis in both *in vitro* and *in vivo* ICH models, improving neurological outcomes (207). Antioxidants such as N-acetylcysteine (NAC) neutralize toxic lipids generated by arachidonate-dependent lipoxygenase 5 (ALOX5) activity, protecting neurons from ferroptosis and improving post-ICH prognosis (208). Another small molecule, HET0016, inhibits the biosynthesis of 20-hydroxyeicosatetraenoic acid (20-HETE), a metabolite of arachidonic acid, and demonstrates neuroprotective effects after ICH (209). Liproxstatin-1, a spiroquinoxalinone derivative, suppresses ferroptosis by reducing ROS and activating the Nrf2/HO-1 pathway, restoring memory function, reducing brain atrophy, and alleviating neurological deficits (210).
3. Antioxidant enhancers: The GPX4–GSH axis is one of the most studied antioxidant pathways in ferroptosis. During acute ICH, GSH treatment enhances antioxidant capacity, reduces brain infarct size, improves neurological deficits, and lowers mortality in ICH mice (211). As an upstream precursor of GSH, NAC partially exerts its protective effects through GPX4–GSH restoration, counteracting ALOX5-mediated lipid peroxidation and neuronal damage (208). Selenium, a trace element essential for GPX4 function, modulates GPX4 expression and activity, thereby blocking ferroptosis and improving brain function post-ICH (212). Natural compounds such as baicalin, curcumin, epicatechin, and isoureguanidine alkaloids have shown promise in ICH models by upregulating GPX4 or SLC7A11 and enhancing cellular antioxidant defenses (210–213). For example, curcumin nanoparticles exert anti-ferroptotic effects via the NRF2/HO-1 pathway (213). EC protects against early-stage hemorrhagic injury by enhancing Nrf2-dependent antioxidant responses and reducing HO-1 induction and iron deposition via Nrf2-independent mechanisms (214). Isoureguanidine alkaloids target the miR-122-5p/TP53/SLC7A11 axis, upregulating miR-122-5p and SLC7A11 while suppressing TP53 to prevent ferroptosis (215). Baicalin inhibits SLC11A2, a key iron transporter, thereby reducing iron deposition and conferring protection in ICH models (216).

In summary, ferroptosis is a key pathological mechanism in ICH, contributing to neuronal death and secondary brain injury. Recent studies have identified multiple molecular targets and therapeutic strategies that inhibit ferroptosis, including iron chelators, lipid peroxidation inhibitors, and natural antioxidants capable of restoring GPX4 and GSH levels. However, the translational gap between basic research and clinical application remains unresolved. In the field of biomarkers, the challenge extends beyond a simple lack of specificity to a more fundamental flaw in translational logic. The low detection accuracy of *in vivo* imaging, caused by interference from free iron, parallels the translational paradox seen in clinical trials of iron chelators such as deferoxamine—effective in animals but ineffective in humans—revealing that a coordinated validation system linking molecular targets, imaging evaluation, and clinical efficacy is still lacking.

Although traditional Chinese medicine research has identified several signaling pathways that regulate ferroptosis, over 90% of these studies are confined to rodent models, without validation of targets or dosage in human patients. This limitation further fragments biomarker translation. In the study of cell death interactions, the problem of “mechanistic isolation” is prominent. The lack of understanding of the signaling cascade between pericyte ferroptosis and endothelial necrosis has made it difficult to select effective vascular-protective targets. If the process is mediated by inflammatory factors, interventions should focus on the TLR4/NLRP3 axis; if driven by iron diffusion, the emphasis should shift to intercellular iron transport channels such as ferroportin. These two mechanisms require entirely different therapeutic approaches. Moreover, the unclear regulatory relationship between the H3K14la/PMCA2 axis and microglial pyroptosis further undermines the theoretical foundation for targeting the integrated “neurovascular–immune” cell death network.

Future research should focus on an integrated strategy that connects mechanism, biomarker, and intervention. The innovative framework proposed in this review provides a starting point for such an approach. At the biomarker level, it is necessary to establish a combined plasma exosome panel adjusted for ethnic variability to minimize individual differences, and to define stratified thresholds according to ICH subtype (hypertensive or traumatic). At the mechanistic level, spatial single-cell transcriptomic studies are urgently needed to map cell death interactions across the hematoma core, penumbra, and surrounding normal tissue. At the interventional level, combination therapy strategies—borrowed from cancer research—should be employed, together with targeted drug delivery systems such as modified nanocarriers to expand the therapeutic window. Only through such coordinated advances can the current gap between mechanistic understanding and clinical efficacy be bridged, enabling ferroptosis modulation to become a truly transformative direction in ICH treatment.

### 4.3 Ferroptosis in traumatic central nervous system injury

TBI and spinal cord injury (SCI) are major contributors to global morbidity and mortality, particularly among young adults. Both conditions involve complex pathological mechanisms that include primary mechanical damage followed by secondary injury cascades involving inflammation, oxidative stress, and regulated cell death pathways such as ferroptosis (217, 218). The progressive loss of neurons and glial dysfunction following TBI or SCI leads to the formation of cavities within the CNS parenchyma, surrounded by dense glial scars, which hinder functional recovery (217).

#### 4.3.1 Ferroptosis in traumatic brain injury

Traumatic brain injury is a leading cause of disability and death among adolescents and young adults globally. It is characterized by rapid neuronal death and extracellular matrix degradation post-injury, culminating in cavity formation and long-term neurological deficits (217, 218). Approximately 50 million people worldwide suffer from TBI annually, imposing substantial burdens on patients, families, and society (219). Moreover, TBI increases the risk of developing neurodegenerative diseases such as Alzheimer’s disease, Parkinson’s disease, and chronic traumatic encephalopathy (220–222). The pathophysiology of TBI includes both primary and secondary injuries, with the latter being amenable to therapeutic intervention. Reducing secondary neuronal death has become a central focus of current treatment strategies (223, 224), and modulating ferroptosis is emerging as a promising approach for neuroprotection after TBI (18). DFO, an iron chelator, exerts neuroprotective effects by reducing intracellular iron accumulation and suppressing the upregulation of ferritin heavy chain (FTH) and light chain (FTL), thereby alleviating local tissue deficits and cognitive impairments in rat models of TBI (225). Fer-1, a synthetic antioxidant, inhibits lipid peroxidation and ferroptosis. Xie et al. demonstrated that intraventricular administration of Fer-1 in TBI mice significantly reduced iron deposition, degenerative neuron numbers, and lesion volume, while improving long-term behavioral outcomes (226). Lip-1, a radical-trapping agent, activates GPX4 and suppresses ferroptosis. Rui et al. showed that Lip-1 downregulates ferroptosis-related proteins and transcripts after trauma, prevents GSH depletion, reduces lipid peroxidation and iron accumulation, decreases neuronal death, and improves motor performance in TBI models (114). Melatonin, a hormone secreted by the pineal gland, reduces iron deposition and neuronal death after TBI, thereby improving neural function. Studies have shown that melatonin provides weak protection in FTH-knockout mice, suggesting its neuroprotective effects are mediated through inhibition of FTH-dependent ferroptosis (114). Xiao et al. further revealed via RNA sequencing that melatonin suppresses ferroptosis by attenuating endoplasmic reticulum stress and lipid peroxidation (227). MicroRNA miR-212-5p, found at low levels in extracellular vesicles from injured brain tissues in TBI mice, has been identified as a negative regulator of PTGS2—a key mediator of ferroptotic signaling—suggesting that miR-212-5p may confer neuroprotection by suppressing ferroptosis (227). Baicalein, a lipoxygenase inhibitor, prevents hippocampal neuronal death induced by mechanical stretch and GPX4 inhibition (e.g., by RSL3). It also reduces phosphatidylethanolamine oxidation and ferroptotic markers in TBI mouse models, thereby improving neurological outcomes (228). Prokineticin 2 (Prok2), a member of the prokineticin family originally isolated from black mamba venom and frog skin, promotes ubiquitination and degradation of ACSL4 by upregulating Fbox10. Intraventricular delivery of AAV-Prok2 enhances GPX4 expression, reduces ACSL4 levels and lesion volume, and improves motor and learning abilities after TBI (229). Picein, a natural compound derived from plants, suppresses ferroptotic responses after TBI—including ferroptosis-related gene expression, iron overload, and lipid peroxidation—and demonstrates greater efficacy than Fer-1 in restoring GPX4 activity (230). Ruxolitinib, a JAK1/2 tyrosine kinase inhibitor approved for myelofibrosis, reverses GPX4 downregulation in TBI and mitigates neurodegeneration, cerebral edema, motor deficits, memory impairment, and anxiety-like behaviors (231). These findings collectively highlight the therapeutic potential of targeting ferroptosis in TBI.

In summary, research on ferroptosis in TBI has progressed from simply identifying mechanisms to actively exploring regulatory strategies. However, in the field of ferroptosis biomarkers, the core challenge has shifted from a lack of indicators to a more fundamental issue—limited clinical applicability. First, current biomarkers (such as serum GPX4) are derived from mouse controlled cortical impact (CCI) models, yet the pathological heterogeneity of human TBI far exceeds that of animal models, making these biomarkers inadequate for guiding clinical treatment. Second, existing biomarkers have not been adjusted for age differences between pediatric and adult TBI patients, leading to high false-positive rates for children in multicenter cohorts. Third, neuroimaging approaches specific to TBI remain unsolved. PET tracers targeting transferrin receptors underperform in diffuse axonal injury, as injury boundaries are unclear and non-specific uptake occurs in perihematomal edema, resulting in signal levels below clinical detection thresholds. Similarly, although fluorescent probes targeting ACSL4—a key regulator of secondary ferroptosis in TBI—achieve specific labeling *in vitro*, they leak out of injured brain tissue *in vivo* due to blood–brain barrier disruption, limiting clinical utility.

At the level of cross-talk between cell death pathways, the temporal evolution of TBI pathology has led to fragmented mechanisms and contradictory findings. On one hand, studies using different animal models—hydraulic impact (vertical force transmission) versus CCI (rotational force transmission)—often produce inconsistent results, highlighting the need to examine how model-dependent differences affect clinical translation. On the other hand, the role of astrocytes in iron transport has been overlooked. After TBI, activated astrocytes downregulate surface ferroportin, leading to intracellular iron retention. Whether this retained iron transfers to adjacent neurons through gap junctions (Cx43), acting as a messenger connecting necroptosis and ferroptosis, remains unknown. This cell-type blind spot has created uncertainty in therapeutic target selection within the TBI cell death network. If iron ions serve as key mediators, strategies should target ferroportin and iron transport; if HMGB1 is central, inhibition of the TLR4/NLRP3 pathway is required. These strategies are entirely different, yet current evidence does not resolve this question. Future studies should prioritize TBI-specific pathology and move beyond the default assumption that animal findings directly translate to humans. A coordinated “mechanism–biomarker–intervention” framework is needed, and this review provides a roadmap for such progress. For biomarkers, a three-dimensional exosome-based system should classify ferroptosis subtypes by injury severity, age, and pathological subtype, paired with dynamic monitoring at 6, 12, 24, and 48 h post-injury to guide therapeutic timing. For mechanisms, single-cell spatial transcriptomics from human postsurgical TBI samples should map cell death interactions across primary injury zones (necroptosis-dominant), secondary injury zones (pyroptosis–ferroptosis interaction), and unaffected regions. For interventions, stage-specific combination therapies should be designed for the 6–72-h secondary-injury window. Only through these approaches can the current gap—clear mechanisms but poor clinical translation—be overcome, advancing ferroptosis-targeted therapy as a strategy to improve neurological outcomes in TBI patients.

#### 4.3.2 Ferroptosis in spinal cord injury

Spinal cord injury is a devastating condition marked by significant motor and sensory dysfunction. With over 27 million cases worldwide and approximately 1 million new cases annually, SCI poses a major public health challenge, particularly among individuals under 30 years old due to falls and traffic accidents (232, 233). Pathologically, SCI involves two phases: the primary injury, caused by direct mechanical trauma, and the secondary injury, driven by biochemical cascades including oxidative stress, inflammation, and regulated cell death (234, 235). Excessive ROS generation—such as superoxide anions, hydrogen peroxide, and hydroxyl radicals—is a hallmark of secondary injury, contributing to cellular dysfunction and death (236). Microglia play a pivotal role in post-SCI neuroinflammation, exhibiting dual phenotypes—pro-inflammatory M1 and anti-inflammatory M2—that influence disease progression (237, 238). Despite advances in diagnostics based on clinical examination, imaging, and biochemical markers (239), effective therapies remain limited. Current interventions focus on surgical decompression, reduction of secondary pathology, and steroid use to suppress inflammation and edema (240). Thus, elucidating the molecular basis of SCI is crucial for improving patient outcomes. DFO not only chelates iron but also enhances GPX4, xCT, and GSH expression, reduces gliosis, and restores long-term locomotor function in SCI models (124). Additionally, DFO mitigates iron overload and ferroptosis in cortical motor neurons after SCI, aiding in motor recovery (241). SRS 16-86, a novel ferroptosis inhibitor with high stability in plasma and liver, improves locomotor function by enhancing GPX4, GSH, and xCT levels, while reducing lipid peroxidation, gliosis, and neuronal damage in SCI models (241). Procyanidins, potent free radical scavengers derived from grape seeds, reduce iron content and thiobarbituric acid-reactive substances (TBARS), inhibit ACSL4 and ALOX15 expression, and enhance HO-1, Nrf2, GSH, and GPX4 levels, ultimately improving motor function in SCI mice (242). Fer-1 suppresses glial activation, reduces local iron accumulation, and downregulates ferroptosis-related genes and mitochondrial alterations, supporting functional recovery after SCI (243). Studies show that zinc can activate the Nrf2/HO-1 protective pathway; decrease ferroptotic intermediates such as GSH, SOD, GPX4, ROS, and MDA; and reverse mitochondrial dysfunction and inflammation (243). Lipoxin A4, an endogenous anti-inflammatory mediator, induces neuroprotection and functional improvement after SCI by activating the Akt/Nrf2/HO-1 axis. In primary spinal neurons, lipoxin A4 also suppresses erastin-induced ferroptosis through the same signaling cascade (244, 245). In experimental SCI models, edaravone—a free radical scavenger—upregulates GPX4 and xCT while reducing ACSL4 and ROS levels, indicating its capacity to ameliorate acute ferroptosis in SCI (246).

In summary, accumulating evidence supports a critical role for ferroptosis in secondary injury after both TBI and SCI, driven by iron overload, lipid peroxidation, and dysregulated ferroptosis-associated proteins (18, 114, 225–235). Ferroptosis contributes to neuroinflammatory responses, immune activation, glial reactivity, and cross-talk with other forms of regulated cell death. Compounds targeting ferroptosis—such as iron chelators, lipid peroxidation inhibitors, antioxidants, and radical scavengers—may exert their neuroprotective effects by modulating iron metabolism, lipid oxidation, and redox homeostasis (18, 114, 225–235). Current studies have confirmed the central role of ferroptosis as a new regulatory target for cell death after spinal cord injury, offering a novel theoretical framework for promoting neural recovery. However, key limitations remain. First, the spinal cord injury microenvironment involves cross-regulation between ferroptosis and other programmed cell death pathways, such as apoptosis and pyroptosis. This work has not yet clarified the priority and interaction mechanisms of ferroptosis within this network, limiting precise interpretation of its role across different injury phases—acute inflammatory response, subacute repair, and chronic glial scar formation. Second, current experiments rely on standardized animal models and do not fully reflect clinical heterogeneity, including differences in injury level, severity, and comorbidities and lack validation in human tissue samples. As a result, the clinical translation potential of ferroptosis-based interventions remains theoretical and cannot yet guide clinical trial design. Future work should advance in three directions. First, research should focus on the dynamic coupling among iron metabolism, lipid peroxidation, and cell fate in the spinal cord injury microenvironment. Single-cell sequencing and spatial transcriptomics should be used to define cell-type-specific ferroptosis pathways in neurons, astrocytes, and microglia, and determine their functional contributions at key stages of repair. Second, ferroptosis-modulating tools with precise spatial and temporal control should be developed, such as nanodelivery systems activated by local microenvironmental features (e.g., pH, reactive oxygen species), to avoid off-target toxicity in healthy neural tissue. Third, multicenter clinical sample repositories should be established, and retrospective cohort studies should examine associations between ferroptosis-related biomarkers (e.g., GPX4, ACSL4) and patient outcomes. This will provide evidence to support clinical trials targeting ferroptosis and ultimately enable a full pipeline from basic mechanisms to translational tools and clinical application, ensuring that ferroptosis-based therapies progress from laboratory research to patient care.

### 4.4 Ferroptosis in PD

**Figure 5:**
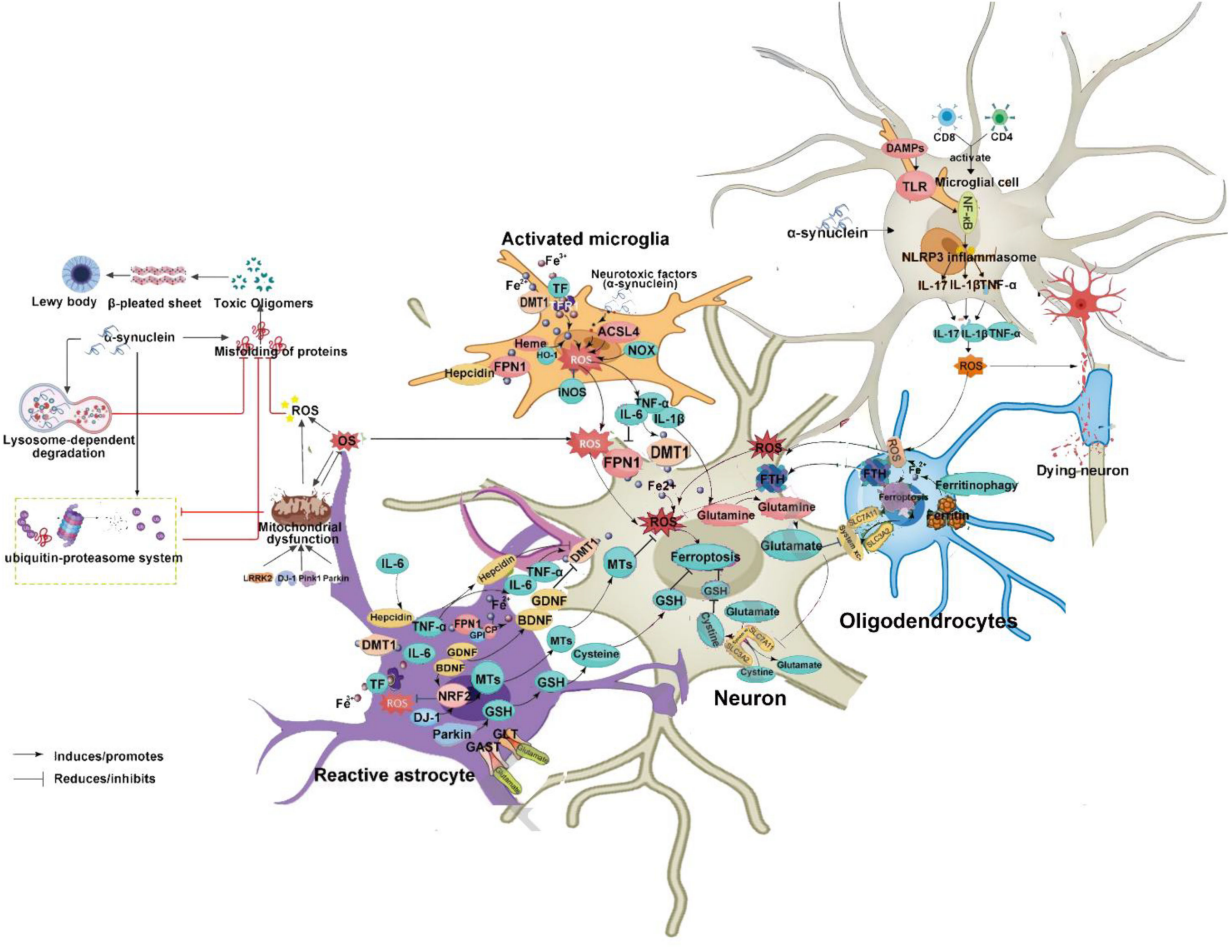
Summary of the mechanisms of ferroptosis in PD. Activation of microglia and astrocytes upregulates DMT1 and downregulates FPN1, leading to iron accumulation in neurons. In contrast, activated astrocytes secrete BDNF and GDNF, which downregulate DMT1 expression and reduce neuronal iron accumulation. Additionally, ROS released by activated microglia promote oxidative stress in neurons. Upregulation of Nrf2 and the release of metallothioneins in astrocytes help protect neurons against oxidative damage. DMT1, divalent metal transporter 1; FPN1, ferroportin 1; BDNF, brain-derived neurotrophic factor; GDNF, glial cell line-derived neurotrophic factor; ROS, reactive oxygen species; Nrf2, nuclear factor erythroid 2-related factor 2; HO-1, heme oxygenase-1; iNOS, inducible nitric oxide synthase; IL, interleukin; TNF, tumor necrosis factor.

PD is a progressive neurodegenerative disorder characterized by motor symptoms such as bradykinesia, resting tremor, rigidity of the trunk and limbs, and a range of non-motor features (247). Although most cases are sporadic, 5%–10% are associated with genetic or aging-related factors. PD affects approximately 1.5%–2.0% of individuals over 60 years of age and up to 4.0% of those over 80 (247). The pathological hallmarks of PD include selective degeneration of dopaminergic neurons in the substantia nigra (SN) and α-synuclein (α-syn) aggregation, and increasing evidence implicates ferroptosis—a form of iron-dependent regulated cell death—in disease progression (248). In PD patients, excessive iron deposition within the SN contributes to the loss of dopaminergic neurons. α-Syn interacts with Fe²^+^ and Fe³^+^, promoting lipid peroxidation and ROS generation, which accelerate oxidative stress and ultimately drive ferroptotic neuronal loss (248). Postmortem brain imaging and histopathological analysis further support this link: elevated iron levels in the SN and globus pallidus have been detected using high-field MRI and quantitative susceptibility mapping (248). These brain regions also show reduced GSH levels and increased lipid peroxidation (LPO)—both of which are key signatures of ferroptosis (248). This suggests that ferroptosis plays a crucial role in explaining the interplay between iron overload, oxidative stress, and selective dopaminergic neuron loss in PD, reinforcing its relevance in PD pathogenesis (**Figure 5**).

#### 4.4.1 Pathogenic mechanisms linking ferroptosis and PD

Iron dyshomeostasis is a central trigger for ferroptosis and closely linked to PD pathology. Iron serves essential roles in oxygen transport, DNA synthesis, mitochondrial respiration, and neurotransmitter metabolism in neurons (249). However, in PD, abnormal iron accumulation in glial cells and dopaminergic neurons has been observed, with iron content strongly correlated with disease severity (249). Studies using high-field MRI and postmortem human brain tissues further confirm that excess iron destabilizes the labile iron pool, enhances ROS production via Fenton chemistry, disrupts respiratory chain function, alters bioenergetics, and promotes protein, DNA, and lipid damage through oxidative stress (250, 251). Intracellular iron homeostasis is maintained by iron metabolism-related proteins such as TfR1, FPN1, FTH1, and FTL. These proteins regulate iron influx and efflux to maintain redox balance (249). In PD, impaired regulation of these proteins leads to increased intracellular iron uptake and decreased export, exacerbating oxidative stress and ferroptosis (252). Moreover, α-syn functions as an iron-reducing agent and can chelate transition metals. Mutant or overexpressed α-syn forms toxic aggregates—Lewy bodies—which promote lipid peroxidation and induce ferroptosis (61). Exposure of neurons expressing familial A53T α-syn to iron accelerates Lewy body formation. Subsequent membrane-associated interactions enhance lipid peroxidation and amplify neuronal injury (61). ROS-induced lipid peroxidation generates MDA and 4-HNE—reactive aldehydes that modify proteins and DNA, serving as mutagenic agents and markers of oxidative damage (253). While 4-HNE may act as a signaling molecule under physiological conditions, at high concentrations, it becomes cytotoxic, inducing mitochondrial dysfunction, proteasomal impairment, and endoplasmic reticulum stress (254). Mounting evidence supports the idea that lipid peroxidation is a common mediator of cellular dysfunction across multiple neurodegenerative diseases (255). Under normal conditions, GSH and GPX protect neurons from ROS and LPO toxicity. The GSH/GSSG ratio serves as a critical antioxidant defense mechanism against lipid ROS and ferroptosis (256). Cysteine, a limiting precursor for GSH synthesis, is transported into neurons via the system Xc^−^ (SLC7A11/SLC3A2), making this pathway a key regulator of redox homeostasis and ferroptosis sensitivity (257). Compounds such as erastin and sulfasalazine, which inhibit system Xc^−^, reduce cysteine availability, compromise GSH synthesis, and decrease GPX4 activity, thereby triggering ferroptosis (258). These findings highlight the functional overlap among iron metabolism, lipid peroxidation, and amino acid dysregulation in PD and suggest that maintaining cellular redox balance and controlling LPO are essential for preventing ferroptosis and delaying PD onset (248).

#### 4.4.2 Role of iron, α-syn, and neuromelanin in mitochondrial metabolism and PD pathogenesis

PD is a multifactorial disease influenced by both environmental and genetic risk factors (259). More than a dozen genes, including *SNCA*, *LRRK2*, *GBA*, *PINK1*, *PRKN*, *DJ-1*, and *ATP13A2*, have been implicated in PD pathogenesis, many of which are directly involved in mitochondrial function and ROS detoxification (260–262). Among these, autosomal dominant forms include mutations in *SNCA* (PARK1), *UCHL1*, *LRRK2* (PARK8), *CHCHD2*, *GBA*, and *VPS35* (PARK17), whereas autosomal recessive forms involve *PARK2* (parkin), *PINK1* (PARK6), *DJ-1* (PARK7), and *ATP13A2* (PARK9) (260). Notably, the LRRK2 G2019S mutation accounts for ~5%–6% of familial and ~1%–2% of sporadic PD cases (261). Environmental toxins such as rotenone and paraquat, which cross the blood–brain barrier, impair electron transport chain function and increase ROS production (262). Additionally, iron itself catalyzes ROS generation via the Fenton reaction, where radicals attack carbon–carbon double bonds in PUFAs, initiating a chain reaction of lipid peroxidation (263). Neuromelanin (NM), a dark pigment present in dopaminergic neurons of the SN, increases during development and stabilizes in midlife but declines after 60 years of age (264). NM acts as a metal chelator, binding Fe²^+^ and Fe³^+^ to suppress Fenton-driven hydroxyl radical formation and dopamine oxidation. However, in PD, excessive iron overwhelms NM’s buffering capacity, leading to free iron buildup, elevated ROS, and subsequent neuronal damage (265). Extracellular NM released from degenerating neurons is phagocytized by microglia, activating inflammatory responses and propagating neurodegeneration (265). Intracellularly, elevated ROS and iron levels can impair NM’s protective effects, accelerating lipid peroxidation and ferroptosis. Thus, NM plays a dual role—protective in healthy neurons and potentially harmful when degraded or released extracellularly (266–270). In summary, the interplay between iron, α-syn, ROS, and NM forms a toxic feedback loop that perpetuates oxidative stress, impairs iron homeostasis, and promotes ferroptosis and neurodegeneration (268). This complex interaction highlights ferroptosis as a central mechanism linking various PD-related pathogenic processes.

#### 4.4.3 Iron chelators as ferroptosis inhibitors in PD therapy

Targeting ferroptosis through iron chelation represents a promising therapeutic approach in PD. Ferroptosis has been confirmed as a prevalent mode of cell death in both *in vitro* and *in vivo* models, and specific inhibition of this process offers significant neuroprotection (271). Clinically used iron chelators such as DFO, deferiprone, and liproxstatin-1 have demonstrated efficacy in slowing disease progression. A placebo-controlled, double-blind trial of DFO in early-stage PD showed reduced iron accumulation in the SN and delayed motor symptom progression (271). In toxin-induced PD models (e.g., MPTP or MPP^+^), these inhibitors protect dopaminergic neurons by suppressing iron-mediated ROS formation, reducing lipid peroxidation, and restoring mitochondrial and synaptic integrity (271, 272). In the MPTP mouse model, MPTP is metabolized to MPP^+^ in glial cells and accumulates in dopaminergic neurons via the dopamine transporter, causing selective neurodegeneration and oxidative stress. Administration of liproxstatin-1 significantly reduces iron-induced damage and improves striatal dopamine levels and motor performance (272). Similarly, in PC12 cell models treated with MPP^+^, increased ROS, elevated MDA and Fe²^+^ levels, and disrupted GSH/GSSG balance reflect the hallmark features of ferroptosis (273). DFO ameliorates these changes by reducing mitochondrial ROS, protecting DA neurons, and inhibiting ferroptosis (273). In chronic MPTP-treated primates, clioquinol—a metal-chelating agent—reduces SN iron and ROS levels, attenuates 4-HNE accumulation, and protects DA neurons (274), indicating that iron chelation can serve as a viable strategy for PD intervention by targeting ferroptosis.

#### 4.4.4 Targeting the Nrf2–GPX4 signaling pathway to inhibit ferroptosis in PD

Nuclear factor erythroid 2-related factor 2 (Nrf2) functions as a master regulator of cellular antioxidant defenses and plays a direct role in modulating GSH biosynthesis, enhancing oxidative stress resistance, and suppressing ferroptosis (275, 276). Accumulating evidence shows that activation of Nrf2 upregulates key genes involved in redox homeostasis, including SLC7A11, glutathione synthetase, and glucose-6-phosphate dehydrogenase, thereby increasing the production of reduced cofactors such as NADPH, GSH, and CoQ10, which collectively enhance cellular antioxidant capacity and counteract disease-associated oxidative stress (277). Opioid peptides such as endorphins and enkephalins, acting via δ-opioid receptors (δ-DORs)—which are highly expressed in the striatum—have also been implicated in neuroprotection. In both PC12 cells treated with statiniron-1 and MPTP-induced PD mouse models, activation of δ-DORs was found to promote Nrf2 expression, reduce accumulation of lipid peroxidation markers MDA and 4-HNE, and preserve mitochondrial structure and function (278). These findings indicate that δ-DORs exert neuroprotection by activating the Nrf2 pathway, thereby inhibiting MPTP-induced ferroptosis and offering a novel mechanism for targeting iron-dependent neuronal death. Collectively, these studies suggest that therapeutic strategies based on the Nrf2–GPX4 signaling axis may open new avenues for PD treatment, providing a multitargeted approach to counteract oxidative damage and ferroptotic cell death.

#### 4.4.5 Modulation of the FSP1–CoQ–NADH pathway to inhibit ferroptosis in PD

FSP1, originally identified as apoptosis-inducing factor mitochondria-derived 2 (AIFM2), is an NAD(P)H-dependent ubiquinone reductase that functions independently of the canonical GSH–GPX4 axis (279). By reducing CoQ10 to its active form CoQ10H_2_, FSP1 serves as a radical-trapping antioxidant that suppresses lipid peroxidation and mitigates iron overload. Additionally, FSP1 acts as a vitamin K reductase, catalyzing the formation of VKH_2_, which further protects against ferroptosis (126). In PD patients and experimental models, FSP1-mediated reduction of CoQ10 significantly decreases ROS generation, reinforcing mitochondrial and lipid membrane integrity (280). These data support the notion that exogenous CoQ10 supplementation can suppress lipid peroxidation, protect dopaminergic neurons, and inhibit α-synuclein aggregation in PD models (281), highlighting the therapeutic potential of CoQ10-based interventions. Moreover, the structural similarity between vitamin K and ubiquinones suggests overlapping mechanisms of action, wherein FSP1-generated VKH_2_ can mimic CoQ10H_2_ in blocking ferroptosis *in vitro* (126). These findings offer innovative directions for drug discovery and therapy development targeting the FSP1–CoQ–NADH pathway in PD.

Recent studies have moved beyond the traditional oxidative-stress-centered view and established ferroptosis as a core regulator of dopaminergic neuron loss in PD, offering a new framework to explain the pathological chain linking iron accumulation, protein aggregation, and neuronal degeneration. However, several fundamental gaps remain. First, a toxic loop involving ferroptosis, α-synuclein, and neuromelanin exists in the PD microenvironment, yet this study has not clarified the bidirectional regulation between ferroptosis and α-synuclein oligomers—specifically, whether oligomers disrupt LIP homeostasis to trigger ferroptosis, or how lipid peroxidation products from ferroptosis accelerate protein aggregation. As a result, the key drivers of PD progression remain incompletely defined. Second, the upstream dominance of ferroptosis within the PD cell death network is not fully validated. Although prior work indicates that ferroptosis acts as a central hub linking reactive oxygen species to autophagy and apoptosis, this study does not establish its priority within the cascade of oxidative stress, neuroinflammation, and multimodal cell death, particularly regarding microglial ferroptosis and its contribution to secondary neuronal injury. Third, clear barriers exist to clinical translation. Current experiments rely on rotenone or MPTP models, which do not replicate environmental exposure patterns or genetic heterogeneity in sporadic PD, and lack evaluation of blood–brain barrier penetration and lesion-targeting delivery. These limitations hinder the transition of ferroptosis-based strategies from invertebrate and rodent models to clinical settings. Future research should advance in three directions. First, at the mechanistic level, single-cell spatial transcriptomics and metabolomics should be used to map ferroptosis pathways in nigral dopaminergic neurons, astrocytes, and microglia, with emphasis on the interplay between NCOA4-mediated ferritinophagy and α-synuclein degradation, to build a dynamic model linking iron dysregulation, proteotoxic stress, and cell death. Second, for translational tool development, multifunctional delivery systems should be optimized. These include RVG29-mediated brain-targeting peptides to cross the blood–brain barrier, pH/ROS-responsive carriers for lesion-specific drug release, and mitochondrial-targeting modules to regulate organelle iron homeostasis, thereby minimizing hematologic toxicity caused by non-specific iron chelation. Third, at the clinical interface, multicenter PD biobanks and retrospective cohorts should be established to validate the association of GPX4, ACSL4, and related biomarkers with motor progression and cognitive decline. In addition, combined clinical trials of ferroptosis inhibitors with exercise interventions should be conducted to assess synergistic effects between pharmacologic and non-pharmacologic strategies. Collectively, these efforts will support a full transition from mechanistic insight to precise therapeutic intervention and accelerate the application of ferroptosis-targeted approaches in PD treatment.

### 4.5 Ferroptosis in AD

AD is the most common form of dementia among the elderly, characterized by progressive cognitive decline, behavioral abnormalities, and loss of social functioning (282). With the global aging population, AD prevalence has been steadily increasing—approximately 3%–7% of individuals over 65 years are affected, with risk nearly doubling every 5 years; in those over 85, prevalence may reach 20%–30% or higher (283).

AD pathogenesis involves complex molecular and cellular mechanisms. Its neuropathological hallmarks include amyloid-beta (Aβ) plaque deposition, NFTs composed of hyperphosphorylated tau protein, cerebral amyloid angiopathy, neuroinflammation, and progressive neurodegeneration (284). The neuroimmune system also plays a role through interactions involving the glymphatic system, resident microglia, astrocytes, and cytokine signaling (285). Emerging studies suggest that iron dyshomeostasis contributes to AD pathophysiology (286, 287). In both human AD brains and mouse models, markers of ferroptosis—such as iron accumulation, glutamate excitotoxicity, and lipid ROS buildup—are frequently observed (288). Compared to controls, AD patients exhibit elevated iron levels in brain regions such as the hippocampus, cortex, and basal ganglia, and these increases correlate with Aβ burden (285–287). Intramuscular administration of the iron chelator DFO has been shown to slow cognitive decline in AD patients (282), while high concentrations of MDA and 4-HNE—key lipid peroxidation products—and reduced expression of GPX4 have been detected across multiple brain regions in AD (285).

**Figure 6:**
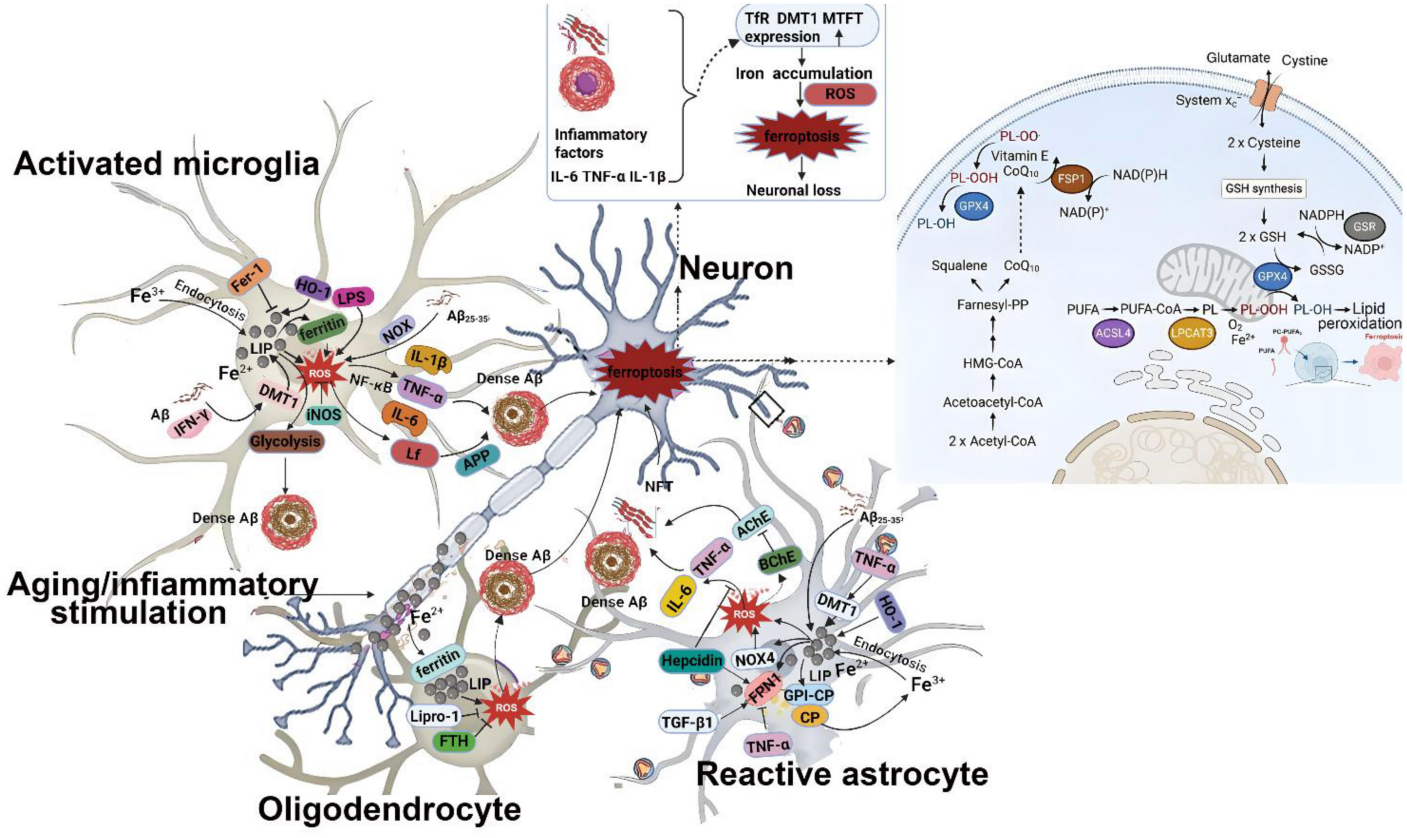
Summary of the mechanisms of ferroptosis in AD. In the pathological context of AD, glial cells are exposed to elevated levels of iron, LPS, and extracellular Aβ released from damaged neurons. i) In microglia, the expression of DMT1 and ferritin is upregulated, leading to increased intracellular iron storage. Continued iron uptake contributes to the formation of the LIP, promoting lipid peroxidation. Iron-induced ROS production activates pro-inflammatory signaling and cytokine release, which exacerbates neuroinflammation, facilitates infiltration into Aβ plaques, and enhances neuronal iron accumulation. Elevated iron levels also promote a shift toward glycolytic metabolism in microglia, reducing phagocytic capacity and accelerating Aβ deposition. Extracellular Aβ enhances ROS production via NOX, further upregulating DMT1 and iron storage, thereby establishing a vicious cycle among iron, ROS, and Aβ. ii) In astrocytes, high iron levels induce a reactive phenotype, promoting ROS generation and inflammatory cytokine release, which contribute to Aβ aggregation and NFT formation. These inflammatory mediators upregulate DMT1 and downregulate Fpn1, leading to further iron accumulation. Heparin inhibits ROS-mediated cytokine release and restores Fpn1 expression. CP also plays a role in maintaining iron homeostasis by regulating iron import and export dynamics. iii) Activated oligodendrocytes produce ROS that exacerbate neuronal oxidative stress. They increase ferritin expression and intracellular iron storage, while also releasing ferritin heavy chain (FTH) as part of an antioxidant defense mechanism to counteract iron-induced cytotoxicity. Collectively, the three major types of glial cells contribute to disrupted neuronal iron homeostasis in the AD brain. Through cytokine release, plaque infiltration, and NFT formation, they initiate neuronal ferroptosis and ultimately lead to neuronal loss. ROS, reactive oxygen species; HO-1, heme oxygenase 1; DMT1, divalent metal transporter 1; NOX, NADPH oxidase; LPS, lipopolysaccharide; IFN-γ, interferon gamma; TNF-α, tumor necrosis factor alpha; IL-1β, interleukin-1 beta; IL-6, interleukin-6; iNOS, inducible nitric oxide synthase; Lf, lactoferrin; APP, amyloid precursor protein; Aβ, amyloid-β peptide; NFT, neurofibrillary tangle; LIP, labile iron pool; Lipro-1, liproxstatin-1; FTH, ferritin heavy chain; BChE, butyrylcholinesterase; AChE, acetylcholinesterase; TGF-β1, transforming growth factor beta 1; NOX4, NADPH oxidase 4; GPI-CP, glycosylphosphatidylinositol-anchored ceruloplasmin; MTFT, mitochondrial ferritin; Fpn1, ferroportin 1; TfR, transferrin receptor.

The Gpx4BIKO mouse model, in which Gpx4 deletion is induced by tamoxifen after 12 weeks, represents a key tool for studying ferroptosis in AD (289). Hambright et al. showed that Gpx4BIKO mice display spatial learning deficits, hippocampal neurodegeneration, and increased markers of ferroptosis, including lipid peroxidation, ERK activation, and neuroinflammatory responses (289). Notably, dietary vitamin E deficiency exacerbates these effects, whereas treatment with the ferroptosis inhibitor liproxstatin-1 significantly improves neuronal survival and behavioral outcomes (289). Glutamate excitotoxicity, driven by impaired system Xc^−^ function, is another mechanism linking ferroptosis and AD (290). This further supports the hypothesis that redox imbalance and iron-dependent lipid peroxidation contribute to AD pathology (**Figure 6**).

#### 4.5.1 Iron dyshomeostasis in AD pathogenesis

As early as the 1960s, cortical iron accumulation was identified as a pathological feature of AD (291). Subsequent studies have established direct links between free iron, oxidative stress, and neuronal death in AD (291–293). Iron is closely associated with both Aβ plaques and NFTs in AD brains. The amyloid precursor protein (APP) undergoes proteolytic cleavage by β-secretase to produce Aβ, and APP itself interacts with FPN to regulate iron efflux from neurons (294). In APP/PS1 transgenic mice, increased free iron and ferritin levels are found alongside Aβ plaque formation (292). Iron overload promotes Aβ and tau misfolding, and conversely, tau aggregation enhances iron retention, forming a self-reinforcing cycle (293). High-iron diets in rats elevate Aβ deposition and tau phosphorylation, whereas iron chelators can mitigate these effects (295). Tau knockout impairs APP-mediated iron export, suggesting that tau dysfunction disrupts iron homeostasis and exacerbates ferroptotic vulnerability (294). Furthermore, iron modulates APP expression and processing—enhancing BACE1 activity and thereby increasing Aβ1–42 production (296). These findings collectively indicate that targeting iron metabolism could represent an effective strategy for controlling ferroptosis in AD.

#### 4.5.2 Redox imbalance contributes to AD pathology

Maintaining redox homeostasis is essential for neuronal integrity, and disruption of this balance is a central contributor to AD progression. GSH, synthesized from glutamate, cysteine, and glycine via GCL and GS, serves as the major intracellular antioxidant and a substrate for GPX4, a key enzyme in preventing lipid peroxidation (289).

Studies show that Aβ induces GSH depletion in cultured neurons, whereas supplementation with γ-glutamyl-L-cysteinyl-glycine ethyl ester (GCEE) restores intracellular GSH levels and suppresses Aβ-induced neurotoxicity (289). In AD models and postmortem brain samples, GSH levels are consistently reduced, indicating that redox instability contributes to disease progression. Beyond GSH, other antioxidant enzymes such as glutathione S-transferases (GSTs) are dysregulated in AD. GST catalyzes reactions between GSH and toxic lipid aldehydes like 4-HNE, reinforcing the importance of maintaining GSH homeostasis in mitigating neurodegeneration (297). In AD models, GPX4 levels are reduced compared to wild-type controls (298), and GPX4 overexpression inhibits LPO and protects against Aβ-induced cortical neuron damage (299). Conditional deletion of *Gpx4* in neurons leads to hippocampal degeneration, astrogliosis, and cognitive impairments—features that are reversed by ferroptosis inhibition using liproxstatin-1 (289). Similarly, in 5×FAD transgenic mice, GPX4 overexpression improves cognition, reduces neurodegeneration, lowers LPO levels, and decreases Aβ production (300). Collectively, these findings highlight the critical role of redox imbalance in AD development.

#### 4.5.3 Enhanced lipid peroxidation and AD progression

PUFAs’ abundance and localization in neuronal membranes are crucial determinants of ferroptosis susceptibility and likely play a role in AD pathogenesis. Due to the high PUFA content in the brain, neurons are particularly vulnerable to ROS attack, leading to LPO and further ROS generation—a vicious cycle that accelerates neurodegeneration. In AD patients, membrane phospholipids and fatty acids are markedly depleted in brain regions rich in senile plaques (SPs) and NFTs, correlating with elevated LPO levels (39). Notably, LPO products such as MDA, 4-HNE, and acrolein co-localize with Aβ plaques, supporting their mutual enhancement: Aβ promotes LPO, while LPO products enhance APP cleavage and Aβ generation (301). Three major classes of lipid peroxidases—lipoxygenases (ALOXs), cyclooxygenases (COXs), and cytochrome P450 reductase (POR)—are dysregulated in AD (302). Among them, 12/15-LOX activity is significantly upregulated in AD brains, and its inhibition rescues memory and learning deficits in mouse models (303). Elevated LPO products such as MDA and 4-HNE are consistently observed in AD brains (304), and dietary supplementation with deuterated PUFAs (D-PUFAs) prevents LPO and reduces toxic Aβ levels in APP/PS1 mice (305). These findings support the concept that LPO is not only a marker but also a driver of AD pathology and that targeting LPO may offer therapeutic benefits by suppressing ferroptosis.

#### 4.5.4 Therapeutic strategies targeting ferroptosis in AD

Given the strong association between ferroptosis and AD, targeting this pathway presents a promising approach for disease intervention. Disrupted iron homeostasis, along with impaired system Xc^−^ and GPX4 pathways, leads to oxidative stress and accelerates AD progression. Therefore, restoring redox and iron balance through iron chelators and antioxidants offers a novel therapeutic avenue (306).

1. Iron chelation therapy: Iron chelators such as DFO, deferiprone, and deferasirox have demonstrated beneficial effects in preclinical and clinical AD models. DFO was first tested in a trial involving 48 AD patients in 1991, where intramuscular administration over 2 years slowed clinical deterioration (307). Deferiprone crosses the blood–brain barrier more effectively than DFO and reduces anxiety-like behavior and cognitive deficits (307). A phase II clinical trial (NCT03234686) is currently evaluating deferiprone’s potential to delay dementia onset (308).

Banerjee et al. showed that chronic administration of deferasirox in aged rats significantly attenuates brain iron accumulation and transferrin overexpression while reducing amyloidogenic processes (308). Additionally, natural small molecules such as tea polyphenols exhibit iron-chelating properties, although careful dosing is required to avoid hepatotoxicity (309). Systemic iron homeostasis must be preserved during chelation therapy, as excessive iron depletion can lead to anemia and other adverse effects (310). Therefore, low-dose chelation strategies are preferred to maintain neuroprotection without disrupting systemic iron balance. New compounds such as HLA20A, a dual-function agent with iron chelation and acetylcholinesterase inhibition, reduce Aβ aggregation induced by iron (311). Hybrid agents combining tacrine and deferiprone have also been developed to target multiple AD-related pathways (312). Several salicylamide derivatives have been designed to inhibit neuroinflammation, acetylcholinesterase, and metal-induced Aβ aggregation via Fe²^+^, Cu²^+^, and Zn²^+^ chelation (313). These multifunctional agents provide improved efficacy and broader therapeutic coverage in AD.

2. Oxidative stress resulting from an imbalance between ROS generation and endogenous antioxidant defenses is a core pathological mechanism in AD. Long-term oral administration of α-lipoic acid in AD patients over 18 months has been shown to prevent iron overload, reduce lipid peroxidation and inflammation, and slow cognitive decline (314). A multicenter, placebo-controlled trial in MCI and AD patients confirmed that vitamin E supplementation reduces LPO and delays progression from MCI to AD (315). Two well-established ferroptosis inhibitors—ferrostatin-1 and liproxstatin-1, aromatic amine-based antioxidants—effectively neutralize radicals generated by ferrous ions and protect neurons from ferroptosis (316). Their protective effects are mediated through activation of NRF2 and GPX4, highlighting the therapeutic relevance of these pathways in AD.

NRF2, a master regulator of antioxidant gene expression, translocates to the nucleus upon phosphorylation and activates downstream targets such as heme oxygenase-1 (HO-1), SOD, and quinone oxidoreductase-1 (NQO1) (316). NRF2 activation not only enhances antioxidant capacity and mitochondrial function but also regulates iron transporters and ferritin expression, contributing to iron homeostasis (317).

**Figure 7:**
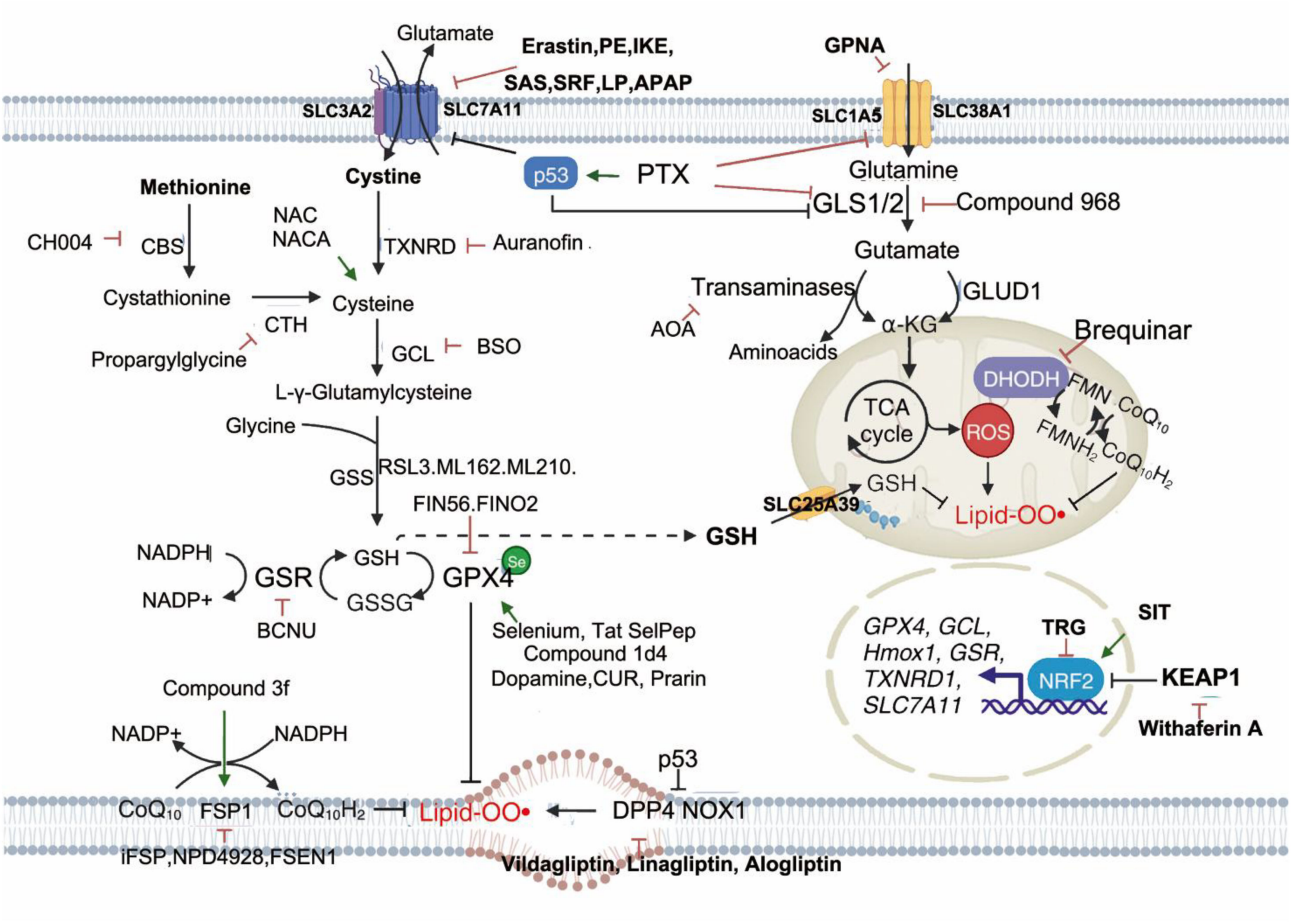
Inhibitory agents targeting ferroptosis-induced oxidative stress. Ferroptosis is closely associated with ROS levels; thus, maintaining redox homeostasis is critical for its regulation. The Xc^−^–GSH–GPX4 axis functions as a primary ROS-scavenging pathway, and various compounds have been designed to modulate ferroptosis by targeting key components of this axis. More recently, the FSP1–CoQ10–NAD(P)H pathway and the mitochondrial DHODH-mediated pathway have emerged as additional regulatory targets of ferroptosis. NRF2 also plays a central role by activating the transcription of genes involved in antioxidant responses, thus serving as a redox-sensitive regulator. Accordingly, targeting the KEAP1–NRF2 axis represents a promising strategy for ferroptosis modulation. Molecules listed in the pink and green boxes act to inhibit or induce the respective redox-regulatory pathways, thereby suppressing or triggering ferroptosis. CBS, cystathionine β-synthase; CoQ10, coenzyme Q10; CTH, cystathionine γ-lyase; DHODH, dihydroorotate dehydrogenase; DPP4, dipeptidyl peptidase-4; FMN, flavin mononucleotide; FMNH_2_, reduced flavin mononucleotide; FSP1, ferroptosis suppressor protein 1; GCL, glutamate–cysteine ligase; GLS, glutaminase; GLUD1, glutamate dehydrogenase 1; GPX4, glutathione peroxidase 4; GSH, glutathione; GSR, glutathione reductase; GSS, glutathione synthetase; GSSG, oxidized glutathione; KEAP1, Kelch-like ECH-associated protein 1; NOX1, NADPH oxidase 1; NRF2, nuclear factor erythroid 2-related factor 2; SLC, solute carrier family; TCA, tricarboxylic acid cycle; TXNRD, thioredoxin reductase.

Numerous preclinical and clinical studies have targeted NRF2 to boost GPX4 activity and counteract ferroptosis. For example, curcumin, a potent NRF2 activator, exhibits ROS-scavenging properties and is being evaluated in multiple AD trials. However, poor bioavailability necessitates improved delivery systems (318). Resveratrol, a natural polyphenol, facilitates NRF2 escape from ubiquitination and is under clinical evaluation for AD, despite limited oral bioavailability (319). Quercetin, a widely distributed flavonoid, stabilizes NRF2 and enhances its transcriptional activity, showing robust antioxidant effects and mitochondrial protection. Clinical data indicate reductions in cerebrospinal fluid (CSF) Aβ and tau following quercetin treatment (320). Liu et al. developed a triphenylphosphonium-modified quercetin that targets mitochondria and effectively chelates excess brain iron, demonstrating the therapeutic potential of plant-derived polyphenolic nanotherapeutics in AD (321). Genistein reduces hippocampal inflammation and upregulates NRF2 in LPS-treated models, improving spatial learning and memory (322). Anthocyanins, when used as dietary supplements in AD mouse models, activate NRF2 and protect neurons from oxidative damage, improving memory function (323). Other reported NRF2 agonists with potential AD-modifying effects include flavonoids (eriodictyol), polyphenols (tannic acid), terpenoids (ginkgolide B), and lignans (forsythiaside A) (324–327) (**Figure 7**).

3. Additional therapeutic approaches: Targeting lipid metabolic disturbances in ferroptosis has also shown promise. Dietary supplementation with ethyl eicosapentaenoate, an omega-3 fatty acid derivative, was assessed in a randomized, double-blind phase III trial involving veterans with mild cognitive impairment. After 18 months, this intervention reduced AD incidence by 50%, potentially by suppressing ferroptosis and LPO (328). Hepcidin, a peptide hormone that regulates systemic iron via FPN binding, can be delivered into the brain to reduce iron deposition and slow AD progression (329). Apolipoprotein E (ApoE) polymorphisms are the strongest genetic risk factors for sporadic AD. Recent imaging studies reveal ApoE–iron interactions, offering new insights into predicting AD risk (330). In summary, Alzheimer’s disease is a complex, multifactorial disorder in which ferroptosis, driven by iron overload, lipid peroxidation, and redox imbalance, plays a pivotal role. Dysregulation of iron metabolism, GSH/GPX4 antioxidant defenses, and lipid oxidation pathways converge to promote neuronal loss and accelerate disease progression. While single-target interventions have shown limited efficacy, emerging strategies—such as iron chelation, NRF2 activation, GPX4 modulation, and multitargeted antioxidants—offer promising avenues for disease modification. Future research should focus on identifying stage-specific ferroptosis regulators, optimizing drug delivery to the CNS, and validating safety and efficacy in large-scale clinical trials. By integrating mechanistic insights with translational approaches, we may develop novel therapies that preserve cognitive function and delay AD onset.

Although current research supports ferroptosis as a central regulator of dopaminergic neuron loss in AD, providing a new mechanistic anchor to link iron accumulation, Aβ aggregation, and tau tangles, key limitations remain. First, the bidirectional regulatory loop between ferroptosis and the Aβ–tau axis in the AD microenvironment has not been resolved. It remains unclear whether Aβ oligomers trigger ferroptosis by suppressing ferroportin and disrupting iron homeostasis, and how lipid peroxidation products generated during ferroptosis (such as 4-HNE) modify tau to accelerate its phosphorylation and aggregation. This gap obscures the core drivers of progressive cognitive decline in AD. Second, the hierarchy within the cell death network requires clarification. Microglial ferroptosis has been shown to amplify neuronal injury by releasing pro-inflammatory mediators, and iron-regulating genes such as SEC24B are upregulated in AD microglia. However, this study does not establish whether ferroptosis acts upstream in the cascade of oxidative stress, neuroinflammation, and apoptosis, nor does it provide evidence for coupling between impaired astrocytic cystine transport and neuronal ferroptosis. Third, critical barriers remain for clinical translation. Most studies rely on transgenic models such as APP/PS1, which do not recapitulate environmental exposures or genetic heterogeneity in sporadic AD, and there is limited evaluation of blood–brain barrier penetration or cognitive benefit for ferroptosis inhibitors such as CuII(atsm). Consequently, ferroptosis-targeted strategies remain at the pathological validation stage. Future work should advance along three directions. First, mechanistic studies should integrate single-cell transcriptomics and spatial metabolomics to map cell-type-specific ferroptosis pathways in hippocampal and cortical neurons and microglia, with particular focus on the link between NCOA4-mediated ferritinophagy and Aβ clearance, thereby building a dynamic model connecting iron dysregulation, proteotoxic stress, and cell death. Second, translational development should focus on dual-functional nanocarriers incorporating Aβ-targeting peptides and RVG29 brain-homing peptides, delivering GPX4 activators or iron chelators with pH/ROS-responsive release to ensure lesion-specific delivery and avoid neurotransmitter synthesis impairment from non-specific iron depletion. Third, clinical efforts should include multicenter AD biobanks to validate associations between biomarkers such as ACSL4 and GPX4 and cognitive scores, as well as combined clinical trials of ferroptosis inhibitors with anti-Aβ monoclonal antibodies to examine therapeutic benefit in antibody-resistant patients. Together, these strategies aim to establish a closed-loop framework linking mechanistic insight, targeted delivery tools, and clinical validation, accelerating the translation of ferroptosis-based interventions toward precision treatment in AD.

### 4.6 Ferroptosis in amyotrophic lateral sclerosis

**Figure 8:**
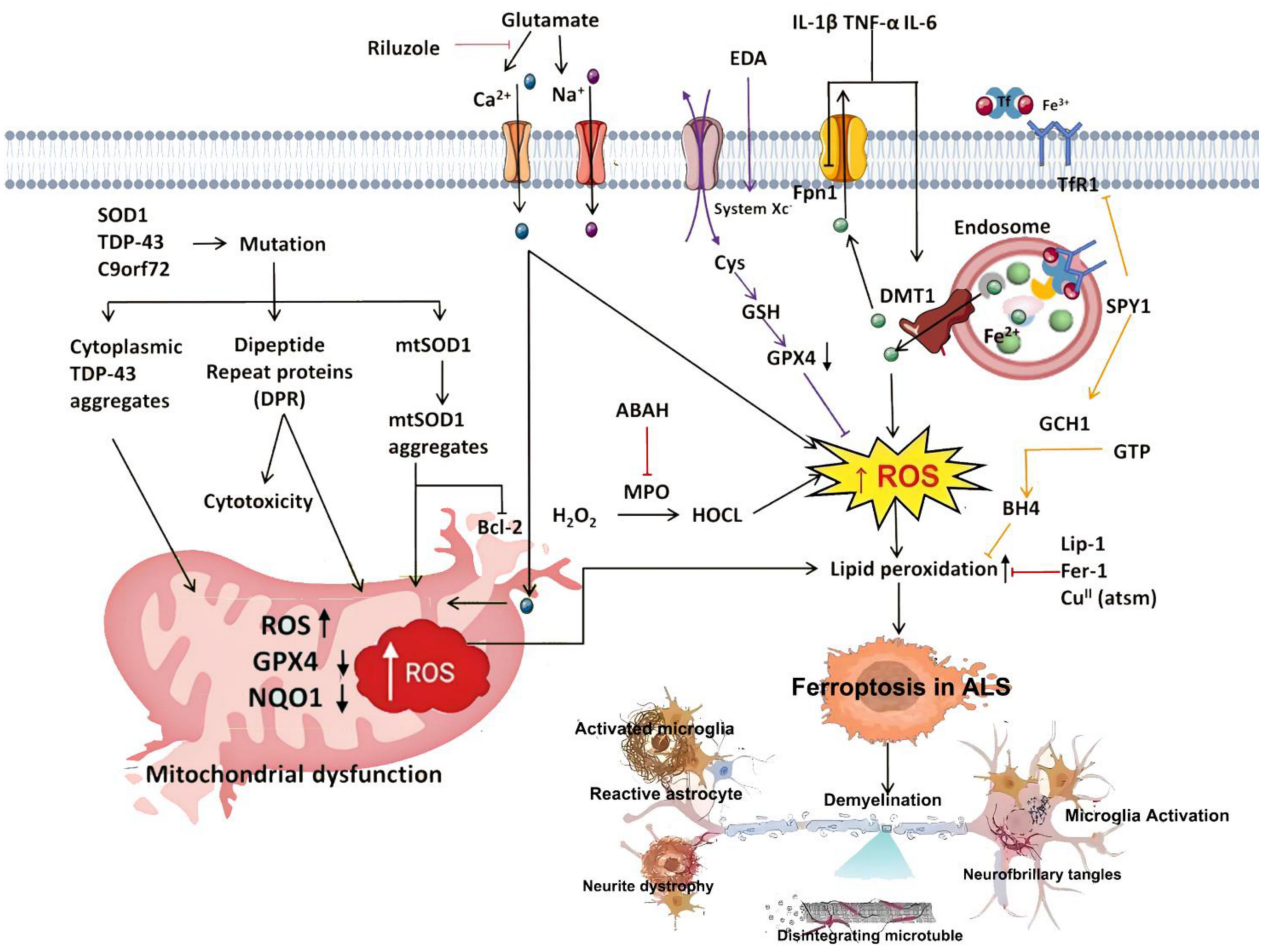
Summary of the mechanisms of ferroptosis in ALS and ferroptosis as a therapeutic target for ALS. Riluzole, a glutamate antagonist, alleviates mitochondrial respiratory chain inhibition in ALS by preventing Ca²^+^ overload and suppressing excitotoxic damage to motor neurons. Edaravone (EDA) inhibits ferroptosis under Cys deficiency and Xc^−^/GPX4 suppression. SPY1 reduces lipid peroxidation (LPO) and inhibits ferroptosis by upregulating the GCH1/BH4 axis and downregulating abnormally elevated TfR1 expression. Within endosomes, Fe³^+^ is reduced to Fe²^+^ and transported into the cytosol via DMT1, increasing the risk of LIP formation and ferroptosis induction. Conversely, Fpn1 helps maintain intracellular Fe²^+^ homeostasis by exporting excess Fe²^+^ from the cell. ABAH, 4-aminobenzoic acid hydrazide; BH4, tetrahydrobiopterin; Ca²^+^, calcium ion; DMT1, divalent metal transporter 1; EDA, edaravone; ERK, extracellular signal-regulated kinase; Fer-1, ferrostatin-1; Fe²^+^/Fe³^+^, ferrous/ferric iron; Fpn1, ferroportin 1; GCH1, GTP cyclohydrolase 1; GPX4, glutathione peroxidase 4; GSH, glutathione; HO-1, heme oxygenase 1; HOCl, hypochlorous acid; Lip-1, liproxstatin-1; LIP, labile iron pool; LPO, lipid peroxidation; MPO, myeloperoxidase; NQO1, NAD(P)H, quinone oxidoreductase 1; ROS, reactive oxygen species; SPY1, Speedy/RINGO cell cycle regulator A; TfR1, transferrin receptor 1; Xc^−^, cystine/glutamate antiporter system.

Iron is the most abundant trace metal in the human brain and plays a central role in various physiological processes, including DNA synthesis, neurotransmitter metabolism, myelination, oxygen transport, and mitochondrial respiration (331). It is primarily stored within ferritin, a heteropolymer composed of heavy (FTH) and light (FTL) chain subunits that together maintain cellular iron homeostasis (331). Disruption of this balance leads to excessive iron accumulation, which catalyzes the Fenton reaction, generating ROS and causing oxidative damage (332). ALS is a rare neurodegenerative disorder characterized by progressive degeneration of upper and lower motor neurons, leading to muscle atrophy, paralysis, and death typically within 3–5 years after symptom onset (333–348). Both clinical patients and animal models—whether in the CNS or peripheral skeletal muscles—show evidence of iron dyshomeostasis, including abnormal iron deposition, altered expression of iron-regulatory proteins, and increased oxidative stress (349–357). These disruptions are associated with disease subtypes, progression rates, and clinical outcomes (334, 335, 338–354, 358), and studies have shown that iron chelation therapy can delay motor neuron degeneration and extend survival in ALS mouse models (352, 355, 358) (**Figure 8**).

#### 4.6.1 Iron metabolism dysregulation in the blood and CSF of ALS patients

Multiple studies and meta-analyses have demonstrated elevated serum ferritin levels in ALS patients compared to healthy controls (334–336, 340). Notably, higher ferritin levels were observed in patients with bulbar-onset, dysphagia, longer disease duration (>12 months), as well as lower scores on the revised ALS Functional Rating Scale (ALSFRS-R) (341). Longitudinal analysis by Devos et al. found that baseline serum ferritin levels negatively correlated with ALSFRS-R scores over 18 months, and these levels progressively increased as disease severity worsened (334). Further stratification based on disease progression rate revealed that fast-progressing ALS patients had significantly higher serum ferritin levels than slow-progressing counterparts (335, 340). However, serum iron levels show variable changes—some studies report increases, others decreases or no change (336, 337, 340, 341). In CSF analyses, Sun et al. found no significant difference in total ferritin between ALS and control groups (340). In contrast, Zheng et al. showed that both FTH and FTL were elevated in CSF from ALS patients, positively correlating with disease progression and inversely with disease duration (339). Patients with lower FTH/FTL ratios exhibited longer survival times. Patti et al. also reported higher CSF iron levels in bulbar-onset ALS compared to spinal-onset cases, particularly in those with disease duration <19 months (338), suggesting that iron metabolism varies across ALS subtypes.

#### 4.6.2 Abnormal iron accumulation in the CNS of ALS patients

MRI studies have identified T2 hypointensity in the motor cortex of ALS patients, which corresponds to pathological findings of iron deposition, predominantly in microglial cells (267, 359). This suggests that neuronal degeneration may lead to local release of iron, which is subsequently scavenged and stored by microglia to maintain brain iron homeostasis (346). Further research has confirmed similar patterns in thalamic regions (344, 360, 361). Li et al. found a positive correlation between cortical iron deposition and upper motor neuron (UMN) involvement (344), while thalamic iron content was inversely related to ALSFRS-R scores (344). However, Yu et al. failed to find associations between T2 signal intensity and disease stage, site of onset, or functional impairment (345), highlighting the need for more refined imaging biomarkers and deeper mechanistic understanding. In terms of clinical subtypes, UMN-predominant ALS shows greater cortical hypointensity compared to LMN-dominant forms, indicating specificity in iron distribution (342, 343). Moreover, Battarai et al. found that ALS patients with lumbar-onset symptoms exhibited more pronounced iron dyshomeostasis in the motor cortex compared to cervical-onset patients (348). Conte et al. proposed that motor cortex sensitivity (MCS) scoring via MRI could serve as an auxiliary diagnostic tool for differentiating ALS-like syndromes, especially when UMN dysfunction is ambiguous (342). Consistent with the findings in human ALS, transgenic mouse models also exhibit iron accumulation, particularly in ventral motor neurons and glial cells of gray and white matter (352, 353, 355). Iron deposits appear early in disease progression and manifest as round cytoplasmic inclusions in motor neurons, with minimal involvement of dorsal structures (352, 355).

This intracellular iron overload is linked to dysregulated expression of key iron-related proteins such as DMT1, TfR1, ferritin, and ferroportin (FPN), which regulate iron influx, storage, and efflux (352, 353, 355, 362). Neuroinflammatory responses during neurodegeneration likely contribute to these disturbances (363). Jeong et al. proposed distinct mechanisms of iron accumulation in neurons versus glial cells: glia primarily accumulate iron through TfR1 and ferritin, whereas neurons rely more on DMT1 and other pathways (352). Additionally, iron deposition correlates with disrupted axonal transport and mitochondrial dysfunction (352, 355). Excess iron in glia promotes TNF-α secretion (355, 364). Administration of iron-clearing agents in ALS mice delays disease onset, reduces ROS production, suppresses TNF-α, and extends survival by preserving motor neuron viability (352, 355).

#### 4.6.3 Motor neuron death and ferroptosis in ALS

Selective motor neuron loss is a hallmark of ALS, and accumulating evidence supports a role for microglia-mediated inflammation as a non-cell-autonomous mechanism accelerating disease progression (365). Emerging studies indicate that ferroptosis contributes to motor neuron death and enhances inflammatory signaling in glial cells (366–373). Ferroptosis is governed by a delicate interplay between pro-oxidant and antioxidant systems. On one hand, iron overload drives ROS generation and lipid peroxidation; on the other hand, the system Xc^−^–GSH–GPX4 axis functions as a critical defense against ferroptotic injury (374). In ALS, multiple lines of evidence support the involvement of this pathway: 1) GSH depletion and reduced GPX4 expression are commonly observed in ALS patients and mouse models (367, 368, 371). 2) Lipid peroxidation markers, such as MDA and 4-HNE, are elevated in ALS tissues (334, 375–377). 3) Downregulation of GPX4 occurs even in presymptomatic stages of disease in mouse models (368, 371). Peng et al. further showed that misfolded Cu/Zn superoxide dismutase 1 (SOD1) activates the myeloperoxidase/hypochlorous acid pathway, leading to GPX4 downregulation and promoting ferroptosis in motor neurons (370). Pharmacological inhibition of GPX4 accelerates motor neuron degeneration and death (366, 367, 369), whereas GPX4 overexpression delays disease onset, improves motor function, and prolongs survival (368, 371). These findings highlight the importance of targeting ferroptosis-associated pathways in ALS treatment and suggest that enhancing GPX4 activity may offer a promising therapeutic strategy.

#### 4.6.4 Microglial activation and ferroptosis in ALS

Microglia, the primary immune cells of the CNS, play dual roles in synaptic regulation and neuroprotection under normal conditions (365). Upon activation, they polarize into either M1 (pro-inflammatory) or M2 (anti-inflammatory) phenotypes, exerting contrasting effects on neuronal survival (365). Growing evidence supports the concept that ALS motor neuron death is a non-cell-autonomous process, where activated microglia amplify neuroinflammation and accelerate disease progression (378–380). Importantly, microglia can directly induce excitotoxicity by releasing glutamate (364, 373). The cystine/glutamate antiporter system Xc^−^, composed of SLC7A11 and SLC3A2, plays a key role in GSH biosynthesis and redox defense. Its activity regulates extracellular glutamate release, influencing microglial polarization states (380). Mesci et al. found that system Xc^−^ expression in microglia affects their M1/M2 status: loss of system Xc^−^ reduces glutamate release, shifts microglia toward the M2 phenotype, and enhances motor neuron survival (373). Similarly, M1-type microglia exhibit reduced susceptibility to ferroptosis compared to M2-type microglia, likely due to high expression of inducible nitric oxide synthase (iNOS) in M1 cells (381–383). Qu et al. showed that iNOS inhibition sensitizes M1 microglia to ferroptosis, reducing their numbers and dampening neuroinflammatory responses (384). As ALS progresses, microglia transition from protective M2 to neurotoxic M1 phenotypes, altering their influence on disease course (365). Exploiting the differential sensitivity of microglial subsets to ferroptosis may offer novel strategies for modulating neuroinflammation and slowing motor neuron loss. In summary, ALS remains a devastating neurodegenerative disease without effective disease-modifying therapies. Emerging evidence implicates iron overload, lipid peroxidation, and dysregulation of the GSH–GPX4 axis in motor neuron loss. Ferroptosis appears to be a key contributor to this pathology, affecting both neurons and glial cells through distinct mechanisms. Targeting ferroptosis via iron chelation, enhancing antioxidant defenses, or modulating microglial polarization may represent viable therapeutic approaches. The use of iron-lowering agents is currently being explored in early-stage clinical trials and warrants further evaluation (385). Given the complex interactions between iron metabolism, oxidative stress, and neuroinflammation in ALS, future studies should integrate multi-omics profiling, longitudinal imaging, and targeted interventions to dissect how ferroptosis influences disease progression. Such efforts may uncover novel strategies for preserving motor neuron integrity and extending patient survival.

Although current studies support ferroptosis as a central regulator of selective motor neuron loss in ALS, providing a new mechanistic anchor to link iron accumulation, protein aggregation, and motor neuron degeneration, several fundamental limitations remain. First, the bidirectional regulatory loop among ferroptosis, pathological proteins, and mitochondrial defects in the ALS microenvironment has not been defined. It is still unknown whether mutant SOD1 fibrils initiate ferroptosis by disrupting mitochondrial iron homeostasis, for example by promoting iron–sulfur cluster release, or how lipid peroxidation products generated during ferroptosis (such as 4-HNE) accelerate TDP-43 nuclear–cytoplasmic mislocalization and aggregation. This uncertainty obscures the key drivers underlying progressive paralysis in ALS. Second, the hierarchy within the cell death network remains unclear. Microglial ferroptosis can activate neurotoxic astrocytes through pro-inflammatory signaling, and ferroptosis-related genes such as ALOX5/15 are highly enriched in microglia, yet this study does not establish whether ferroptosis is upstream in the cascade involving mitochondrial complex IV defects, oxidative stress, and neuroinflammation. Evidence linking impaired astrocytic cystine transport to neuronal ferroptosis is also lacking. Third, major obstacles persist for clinical translation. Current experiments rely on SOD1 transgenic models, which do not recapitulate mitochondrial complex IV deficiency or the genetic heterogeneity of sporadic ALS (such as C9orf72 repeat expansion) and do not assess spinal cord targeting or pharmacokinetic limitations of ferroptosis inhibitors, including low solubility and short half-life. These gaps hinder progress toward clinical application. Future work should advance in three directions. First, mechanistic studies should combine single-cell spatial transcriptomics with mitochondrial metabolomics to map ferroptosis pathways in spinal ventral horn motor neurons, microglia, and astrocytes, with emphasis on the coupling between NCOA4-mediated ferritinophagy and SOD1/TDP-43 clearance, thereby establishing a dynamic model linking mitochondrial iron dysregulation, proteotoxic stress, and cell death. Second, translational tool development should focus on dual-functional nanocarriers incorporating motor-neuron-targeting peptides and RVG29, delivering GPX4 activators or mitochondria-targeted iron chelators with ROS/pH-responsive release to achieve lesion-specific delivery and avoid neurotransmitter-synthesis impairment due to non-specific iron depletion. Third, clinical translation should include establishing multicenter ALS biobanks to validate associations between biomarkers such as ACSL4 and GPX4 and clinical measures including ALSFRS-R scores and survival, and conducting combined trials of ferroptosis inhibitors with mitochondrial protectants to evaluate therapeutic benefit in sporadic ALS. Together, these strategies aim to build an integrated framework spanning mechanistic studies, targeted delivery systems, and clinical validation, ultimately advancing ferroptosis-based therapies toward precision treatment for ALS.

### 4.7 Ferroptosis and Friedreich ataxia

FRDA is a juvenile-onset, autosomal recessive neurodegenerative disorder characterized by progressive limb ataxia, pyramidal tract signs, and dysarthria (386). The disease is primarily caused by deficiency of frataxin, a mitochondrial iron-binding protein essential for iron–sulfur cluster biosynthesis and redox homeostasis. Mutations in *FXN* lead to impaired iron–sulfur cluster formation, triggering intracellular iron starvation responses that paradoxically result in mitochondrial iron overload, oxidative stress, and subsequent cellular dysfunction (386). Studies have shown that treating FRDA lymphoblasts and fibroblasts with hydrogen peroxide (H_2_O_2_) leads to excessive iron and ROS accumulation—effects that can be mitigated by the iron chelator DFO. Moreover, the ferroptosis inhibitor Fer-1 suppresses erastin-induced cell death in B lymphocytes from FRDA patients, suggesting a potential link between ferroptosis and disease pathology (386). However, contrary findings indicate that FRDA-derived B cells exhibit increased resistance to erastin-induced ferroptosis compared to controls (387). Notably, both DFO and ammonium ferric citrate enhance erastin-induced cell death, implying that although systemic iron overload exists in FRDA, cytosolic iron may still be limiting, and iron metabolism is dysregulated, contributing to cellular vulnerability. Further research suggests that LPO plays a role in FRDA progression (388). Inhibitor studies have identified novel oleic acid analogs that protect against neuronal damage in multiple FRDA cell models (389). Quatrana et al. found that 4-HNE, a toxic product of lipid peroxidation, serves as a potential mediator linking ferroptosis and inflammation in FRDA fibroblasts (390). Additionally, frataxin deficiency in dorsal root ganglia disrupts iron homeostasis and impairs Nrf2 activation and its downstream redox signaling pathways, thereby promoting ferroptotic injury (391). Activation of Nrf2 has been proposed as a protective strategy against ferroptosis in FRDA (277). Recently, the FDA approved omaveloxolone, a potent activator of Nrf2 signaling, as the first therapeutic agent for FRDA (392), marking a significant step toward targeting redox imbalance and ferroptosis in this disease. Despite evidence of systemic iron and ROS accumulation in FRDA, whether neuronal loss is directly mediated by ferroptosis remains an open question requiring further mechanistic exploration.

Although current research indicates that ferroptosis plays a central regulatory role in the pathological process of FRDA—linking mitochondrial iron accumulation, neurodegeneration, and cardiomyopathy—and provides a new theoretical anchor for understanding the “FXN deficiency–iron dysregulation–target-organ injury” axis, several fundamental limitations remain. First, the bidirectional regulatory mechanism connecting the core FRDA pathway of “FXN–iron–sulfur clusters–ferroptosis” has not been clarified. It remains unresolved whether reduced frataxin triggers ferroptosis by inhibiting iron–sulfur cluster synthesis enzymes (such as NFS1 and ISCU) and disrupting mitochondrial iron homeostasis, nor is it known how lipid peroxidation products generated during ferroptosis further impair frataxin function and exacerbate iron–sulfur cluster assembly defects. This gap clouds the identification of the key drivers underlying progressive ataxia and cardiomyopathy in FRDA. Second, tissue-specific regulatory mechanisms remain insufficiently understood. Purkinje cells, dorsal column neurons, and cardiomyocytes all exhibit ferroptotic features in FRDA, yet this study does not distinguish pathway differences across organs—for example, whether cerebellar injury depends more on ACSL4-mediated lipid dysregulation and whether cardiomyocyte vulnerability primarily reflects GPX4 inactivation. Moreover, evidence is lacking for coupling between ferroptosis in astrocytes or cardiac fibroblasts and secondary injury to parenchymal cells, limiting interpretation of the coordinated progression of neurologic and cardiac damage. Third, key barriers impede clinical translation. Current experiments rely on FXN-knockdown cells or YG8s mouse models, which do not replicate heterogeneity in GAA repeat expansion length observed in patients (a factor strongly correlated with disease severity) and do not evaluate organ-targeting efficiency or long-term safety of ferroptosis inhibitors such as deferiprone—particularly whether simultaneous delivery to cerebellum and heart is achievable and whether sustained treatment disrupts erythroid iron homeostasis and induces anemia. As a result, therapeutic strategies remain largely at the pathological validation stage.

Future studies should progress along three directions. First, mechanistic work should integrate spatial metabolomics and mitochondrial proteomics to map ferroptosis pathways in cerebellar, spinal, and cardiac tissues from FRDA patients, with emphasis on the interaction between the iron–sulfur cluster synthesis complex (NFS1–ISCU–ISD11) and ferroptosis-related molecules (GPX4, Nrf2). This will support development of a dynamic model linking FXN deficiency, iron–sulfur cluster disruption, and mitochondrial ferroptosis and help identify organ-specific drivers. Second, translational tools should focus on dual-targeted nanocarriers designed to reach cerebellum and myocardium—using TAT peptides for blood–brain barrier penetration and CTP peptides for cardiomyocyte targeting—loaded with mitochondria-directed ferroptosis modulators capable of activating GPX4 while supporting iron–sulfur cluster synthesis, and released in response to GAA-repeat-dependent triggers to avoid off-target iron perturbation. Third, clinical efforts should include establishing multicenter FRDA biobanks to validate associations between ferroptosis biomarkers (such as ACSL4 and mitochondrial iron content) and clinical measures including SARA ataxia scores and left ventricular ejection fraction, and conducting combination trials of ferroptosis inhibitors with iron–sulfur cluster metabolism regulators to evaluate synergistic improvement in neurologic and cardiac function. Collectively, these steps aim to build an integrated framework linking mechanistic understanding, targeted delivery technologies, and clinical validation, advancing ferroptosis-based therapy from FRDA research toward precision treatment.

### 4.8 Ferroptosis in periventricular leukomalacia

Periventricular leukomalacia (PVL) is a secondary white matter disorder predominantly affecting preterm infants, presenting clinically with bilateral spastic paresis, tetraplegia, and cognitive impairment (393, 394). It results from axonal injury and abnormal death of oligodendrocytes, which are highly susceptible to oxidative damage. Currently, no effective treatments exist for PVL, and neuroprotective or antioxidant-based interventions remain experimental with limited clinical validation. In PVL patients, oligodendrocyte death is closely associated with iron accumulation, excessive lipid peroxidation, and elevated levels of ROS, MDA, and non-transferrin-bound iron in the CSF (395), providing a compelling rationale for targeting ferroptosis in disease management. Ferroptosis-specific inhibitors such as Fer-1 have demonstrated efficacy in reversing GSH depletion-induced oligodendrocyte death *in vitro* (396). Skouta et al. showed that Fer-1 suppresses cell death in HD, PVL, and renal failure models by inhibiting LPO without affecting mitochondrial ROS production or lysosomal membrane permeabilization (125). These findings led to the development of improved ferrostatin derivatives, offering new avenues for pharmacological intervention. These data support the hypothesis that ferroptosis contributes significantly to PVL pathogenesis, and modulation of oligodendrocyte ferroptosis represents a promising therapeutic target for future neuroprotective strategies.

In summary, although current studies suggest that ferroptosis plays a central regulatory role in the pathological sequence of PVL—linking hypoxia–ischemia, inflammatory infiltration, and white-matter necrosis—and provides a new mechanistic basis for understanding the pathway from periventricular iron accumulation to oligodendrocyte loss and motor developmental impairment, several essential gaps remain. First, the bidirectional regulatory mechanism within the core PVL pathway of “hypoxia–ischemia–ferroptosis–oligodendrocyte injury” has not been defined. It also remains unclear how lipid peroxidation products generated during ferroptosis impair myelin-forming capacity of oligodendrocyte precursor cells (OPCs) and worsen white-matter softening, leaving the key drivers of progressive motor dysfunction in PVL insufficiently delineated. Second, the hierarchy within the cell death network requires clarification. Microglial ferroptosis has been shown to amplify neurotoxicity by releasing pro-inflammatory mediators (such as TNF-α and IL-1β), and ferroptosis-related genes including ACSL4 and ALOX15 are highly enriched in microglia within PVL lesions. However, current studies have not established whether ferroptosis acts upstream in the cascade involving oxidative stress, astrocyte activation, and multimodal cell death. In particular, evidence is lacking regarding differential sensitivity and molecular mechanisms by which OPCs and mature oligodendrocytes respond to ferroptosis, limiting understanding of the white-matter selectivity characteristic of PVL pathology.

Future research should advance in two primary directions. First, mechanistic studies should integrate single-cell spatial transcriptomics and mitochondrial metabolomics to map ferroptosis pathways in OPCs, mature oligodendrocytes, and microglia within periventricular white matter of infants with PVL. A key focus should be the interaction between the HIF-1α-regulated iron metabolism axis (such as transferrin receptor 1 and ferritin light chain) and ferroptosis-related molecules including GPX4 and Nrf2, to establish a dynamic model connecting hypoxia–ischemia, iron dysregulation, and oligodendrocyte ferroptosis, and to identify stage-specific vulnerability across oligodendrocyte maturation. Second, clinical translation should involve establishing multicenter biobanks for preterm infants to validate associations between ferroptosis biomarkers (such as cerebrospinal fluid ACSL4 levels and MRI-detected white-matter iron deposition) and motor development scores (GMFM and Bayley scales). Prospective trials combining ferroptosis inhibitors with neuroprotective agents (such as edaravone) are also needed to explore whether targeting ferroptosis can aid prevention and treatment of PVL in extremely preterm infants. Collectively, these efforts are expected to build an integrated framework linking mechanistic insight, targeted interventions, and clinical evaluation, advancing ferroptosis-directed strategies from foundational PVL research toward precision neuroprotection in preterm infants.

### 4.9 Ferroptosis in Huntington’s disease

HD is an autosomal dominant neurodegenerative disorder caused by CAG repeat expansions in the *HTT* gene, which encodes mutant huntingtin (mHTT) proteins containing elongated polyglutamine (polyQ) tracts. These misfolded proteins accumulate in neurons, leading to selective degeneration of striatal neurons and progressive motor and cognitive deficits (397). Clinical studies have revealed reduced plasma GSH levels and decreased erythrocytic GPX activity in HD patients compared to age-matched controls (398). Kumar et al. induced HD-like phenotypes in rats using 3-nitropropionic acid (3-NP), observing widespread GSH depletion in the striatum, hippocampus, and cortex (399). In cellular models, GSH depletion increases neuronal death in striatal neurons expressing mHTT, reinforcing the role of GSH-dependent redox imbalance in HD progression (400). Skouta et al. demonstrated that both Fer-1 and SRS11-92, specific ferroptosis inhibitors, significantly enhance survival in striatal neurons expressing mutant huntingtin in a dose-dependent manner, providing initial evidence that targeting ferroptosis may offer therapeutic benefits in HD (401). Recent studies also highlight the role of arachidonate 5-lipoxygenase (ALOX5) in mediating ferroptosis under conditions of oxidative stress in HD (402). Liu et al. further elucidated ferroptosis-related mechanisms in striatal neuron subtypes affected by laid-back ligand-gated channel agonist (laduviglusib), identifying cell-type-specific targets in HD (403).

**Figure 9:**
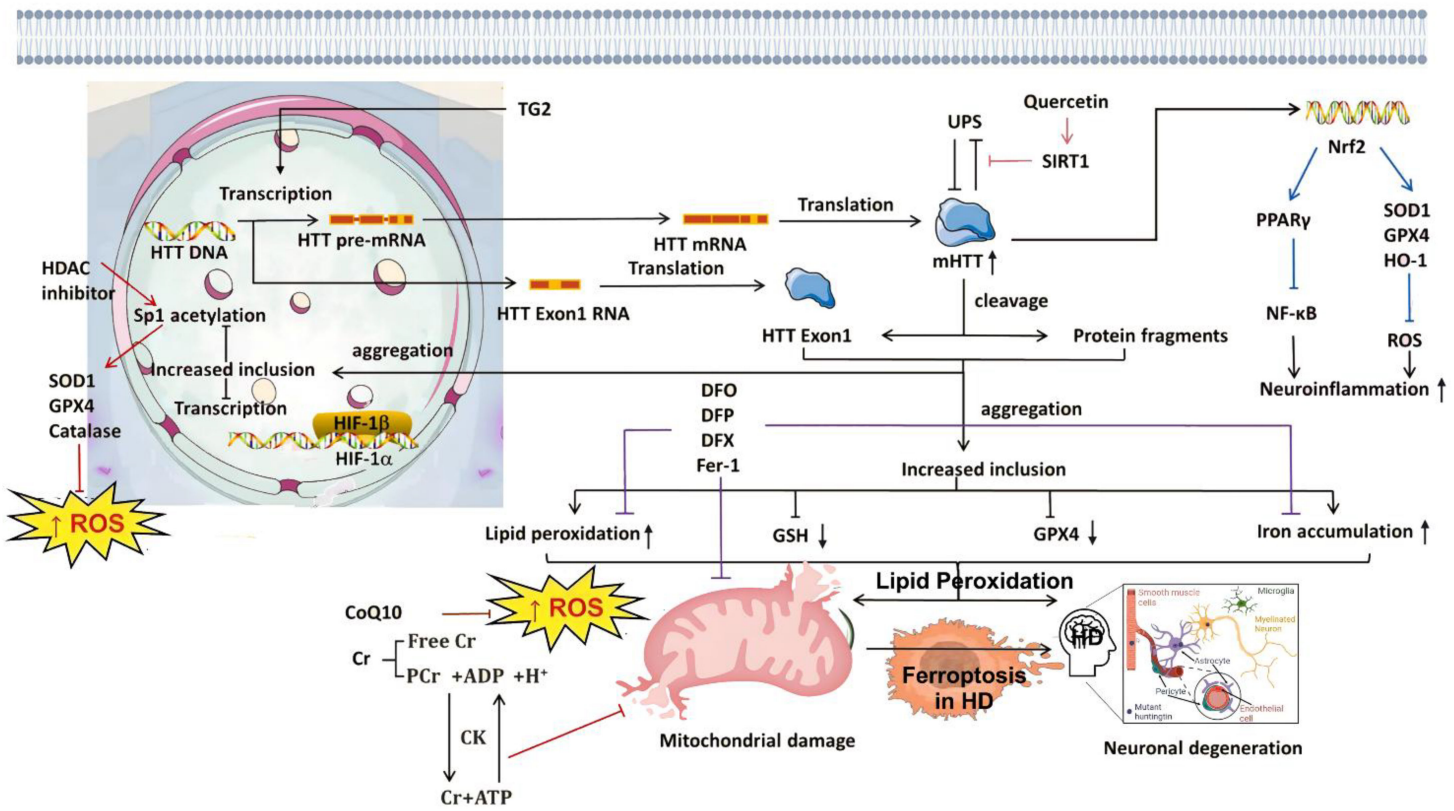
Summary of the mechanisms of ferroptosis in HD and ferroptosis as a therapeutic target for HD. Frequent cleavage of mHTT leads to the formation of toxic aggregates, accompanied by reductions in GSH and GPX4, and increased ionic accumulation, ultimately resulting in mitochondrial damage and neuronal degeneration. Iron chelators such as DFO, DFP, and DFX have been shown to reduce iron overload, attenuate LPO, and improve mitochondrial function. QCT exerts neuroprotective effects in HD by targeting SIRT1, thereby mitigating mHTT-mediated inhibition. In addition, mHTT aggregates induce neuroinflammation by suppressing the Nrf2 signaling pathway. Creatine (Cr), through its active form PCr, participates in the CK-mediated ADP–ATP cycle, alleviating oxidative stress in mitochondria. Together with CoQ10, Cr helps reduce mitochondrial ROS accumulation. HDAC inhibitors enhance the acetylation of the transcription factor Sp1 and upregulate SOD1, GPX4, and catalase, thereby counteracting oxidative stress. HIF-PHD inhibitors promote the expression of HIF-1α, which dimerizes with its β-subunit partner and acts as a transcriptional activator to enhance HIF-1α-dependent gene expression. ADP, adenosine diphosphate; ATP, adenosine triphosphate; CK, creatine kinase; CoQ10, coenzyme Q10; Cr, creatine; DFO, deferoxamine; DFP, deferiprone; DFX, deferasirox; Fer-1, ferrostatin-1; GSH, glutathione; GPX4, glutathione peroxidase 4; HD, Huntington’s disease; HDAC, histone deacetylase; HIF-1α, hypoxia-inducible factor 1 alpha; HIF-PHD, HIF prolyl hydroxylase; LPO, lipid peroxidation; mHTT, mutant huntingtin; Nrf2, nuclear factor erythroid 2-related factor 2; PCr, phosphocreatine; QCT, quercetin; ROS, reactive oxygen species; SIRT1, sirtuin 1; SOD1, superoxide dismutase 1; Sp1, specificity protein 1; TG2, transglutaminase 2; UPS, ubiquitin–proteasome system; PPARγ, peroxisome proliferator-activated receptor gamma.

Together, these findings suggest that ferroptosis contributes to HD pathology, and its inhibition may represent a viable strategy for disease modification (**Figure 9**).

In summary, although current studies demonstrate that ferroptosis plays a central regulatory role in the pathogenic cascade of HD—linking mHTT aggregation, neuroinflammation, and neuronal loss—and provide a new mechanistic basis for understanding the sequence of basal ganglia iron accumulation, striatal atrophy, and motor dysfunction, several fundamental limitations remain. First, the bidirectional regulation within the core HD pathway of “mHTT–ferroptosis–mitochondrial dysfunction” has not been fully defined. It is still unclear whether mHTT initiates ferroptosis by suppressing ferroportin or activating NCOA4-mediated ferritinophagy to disrupt striatal iron homeostasis, nor is it known how lipid peroxidation products generated during ferroptosis (such as 4-HNE) modify mHTT to accelerate its nuclear aggregation and toxic propagation. These gaps obscure the key drivers underlying progressive chorea and cognitive decline in HD. Second, the hierarchical structure of the cell death network remains unresolved. Microglial ferroptosis has been shown to induce neurotoxic astrocytes through pro-inflammatory signaling (such as IL-6 and TNF-α), and ferroptosis-related genes including ACSL4 and GPX4 are highly enriched in microglia within HD lesions. However, current evidence does not establish whether ferroptosis acts upstream in the cascade involving mHTT toxicity, excitotoxicity, and multimodal cell death. Notably, there is insufficient evidence regarding differential susceptibility and molecular mechanisms by which D1- and D2-type medium spiny neurons (MSNs) respond to ferroptosis, limiting understanding of the striatal selectivity characteristic of HD. Third, major barriers remain for clinical translation. Current studies rely on R6/2 or Q175 transgenic mouse models, which do not recapitulate the heterogeneity of CAG repeat length or environmental exposures seen in sporadic HD. In addition, the basal ganglia targeting efficiency and blood–brain barrier penetration of ferroptosis inhibitors (such as Fer-1) have not been evaluated, and long-term safety data in HD patients are lacking, including whether treatment disturbs iron metabolism in unaffected brain regions and causes cognitive side effects. As a result, ferroptosis-modulating strategies remain largely confined to pathological validation.

Future research should advance in three directions. First, mechanistic studies should integrate single-cell spatial transcriptomics and mitochondrial metabolomics to map ferroptosis pathways in MSNs and microglia in the basal ganglia and striatum of patients with HD. Particular focus should be placed on interactions between mHTT, the iron–metabolism axis (such as transferrin receptor 1 and hepcidin), and key ferroptosis mediators (GPX4 and Nrf2) to build a dynamic model linking mHTT aggregation, iron dysregulation, and neuronal ferroptosis, and to define subtype-specific vulnerability of MSNs. Second, translational development should focus on dual-functional nanocarriers designed for striatal targeting and blood–brain barrier penetration—using DARPP-32-binding peptides to target affected MSNs and PEGylation to enhance brain entry—loaded with mitochondria-directed ferroptosis modulators that both activate GPX4 and chelate iron, combined with mHTT-responsive release systems to avoid interference with normal neuronal function. Third, clinical translation should include establishing multicenter HD biobanks to validate associations between ferroptosis biomarkers (such as cerebrospinal fluid ACSL4 levels and striatal iron MRI signals) and clinical measures (UHDRS scores and transcranial motor-evoked potentials). Inspired by the logic of combining HDAC inhibitors with ferroptosis inducers, clinical trials pairing ferroptosis inhibitors with mHTT-clearing agents should be conducted to evaluate therapeutic benefit in patients with mid- to late-stage HD. Collectively, these efforts aim to establish an integrated framework linking mechanistic insight, targeted delivery strategies, and clinical validation, promoting translation of ferroptosis-based interventions from HD research to precision treatment.

## 5 Targeting ferroptosis with small-molecule inhibitors and natural compounds: a dual-track strategy and translational challenges for the treatment of neurodegenerative diseases

Ferroptosis has emerged as a novel pathogenic cell death pathway in neurodegenerative diseases. Its druggable characteristics—iron dependence, lipid peroxidation-driven mechanisms, and the ability to regulate antioxidant systems—make it an attractive therapeutic target. In recent years, two major intervention strategies have demonstrated significant neuroprotective potential in preclinical models: synthetic small-molecule inhibitors (such as ferrostatin-1, liproxstatin-1, and iron chelators) and naturally derived small molecules (such as curcumin, resveratrol, baicalein, and ginsenosides). However, these two classes differ fundamentally in their mechanisms of action, pharmacokinetics, target specificity, safety profiles, and translational pathways. This chapter will systematically review the mechanisms, efficacy, neuro-specific advantages, and limitations of representative compounds and will further explore the unique challenges and future directions of optimizing ferroptosis-targeted therapies in the context of neurodegenerative disease.

Among synthetic small-molecule inhibitors, mechanisms are well-defined and inhibitory potency is strong, but limited blood–brain barrier penetration and potential toxicity remain major obstacles to clinical translation. These inhibitors are typically designed against the core ferroptosis pathway, with clear molecular targets, and represent the most intensively studied tools to date. Lipid peroxidation scavengers/chain reaction blockers act as radical-trapping antioxidants (RTAs), interrupting the chain amplification of lipid peroxidation and protecting membrane phospholipids, particularly PUFA-containing phospholipids, from oxidative damage. Representative examples include Fer-1 and Lip-1. Their advantages include direct action, rapid onset, and low *in vitro* IC_50_ values (nM–μM range). However, several neuro-specific challenges limit their application: 1) poor blood–brain barrier penetration: Despite high lipophilicity, Fer-1 and Lip-1 lack active transport mechanisms, resulting in brain concentrations <10% of peripheral levels and requiring high doses or intrathecal delivery, which restricts clinical use. 2) Low metabolic stability: Both compounds are rapidly degraded by hepatic CYP450 enzymes, with short half-lives (<2 h), making it difficult to maintain effective brain concentrations. 3) Potential off-target effects: At high doses, they may disrupt normal lipid metabolism or mitochondrial function. Future optimization strategies include the development of brain-targeted prodrugs (e.g., ester modifications, peptide conjugation), the design of nanodelivery systems (PLGA, liposomes, exosome encapsulation) to enhance brain accumulation, and the identification of next-generation RTA molecules (such as SRS11–92 and SRS16-86) with improved blood–brain barrier permeability and metabolic stability. Another synthetic strategy involves iron homeostasis modulators (iron chelators), which act by binding free Fe²^+^, thereby blocking the Fenton reaction, reducing hydroxyl radical generation, and preventing the initiation of lipid peroxidation. Representative agents include DFO, DFP, and deferasirox. A major advantage of this class is that some drugs (e.g., DFP) have already been approved by the FDA for systemic iron overload, providing relatively well-established safety data.

In the context of natural small-molecule compounds, their structural diversity, low toxicity, and ability to regulate multiple pathways confer unique advantages in ferroptosis intervention, particularly for the long-term treatment of chronic neurodegenerative diseases. The core strengths of natural compounds include the following: first, multitarget network regulation, enabling simultaneous modulation of iron metabolism, lipid peroxidation, antioxidant systems, and inflammatory pathways, which aligns with the multifactorial pathology of neurodegenerative diseases. Second, they exhibit good safety and tolerability, as many compounds are derived from dietary sources or traditional medicines with low toxicity during long-term use. Third, they provide synergistic anti-inflammatory and neuroprotective effects; for example, curcumin and resveratrol not only inhibit microglial activation but also suppress ferroptosis, thereby disrupting the vicious cycle of “cell death–inflammation”.

Nevertheless, natural compounds also face critical challenges and controversies. One major issue is difficulty in attributing mechanisms: It remains unclear whether their neuroprotective effects arise specifically from ferroptosis inhibition or indirectly through anti-inflammatory, anti-aggregation, or mitochondrial protective pathways, and genetic evidence is lacking (e.g., validation in GPX4 conditional knockout models). Another limitation is extremely low bioavailability: Most polyphenols suffer from poor oral absorption, strong first-pass metabolism, and insufficient brain concentrations (for instance, curcumin reaches less than 1% of plasma levels in the brain). A third problem lies in batch variation and lack of standardization: Plant extracts are compositionally complex, with unstable ratios of active components, making it difficult to establish clear dose–response relationships. Finally, potential bidirectional effects have been observed; certain compounds (such as artesunate and some flavonoids) may either promote or inhibit ferroptosis depending on dosage or microenvironment, creating a narrow therapeutic window. Future directions include structural modifications to enhance brain targeting (e.g., curcumin nanocrystals, resveratrol phosphate prodrugs), identification of core pharmacophores and direct targets using chemical biology techniques (such as ABPP and CETSA), establishment of standardized evaluation systems for “ferroptosis-inhibition potency” to enable cross-comparison of EC_50_ and therapeutic indices across consistent cell and animal models, and the development of combination strategies integrating natural compounds with synthetic inhibitors (e.g., low-dose Lip-1 with curcumin) to reduce dosage, minimize side effects, and enhance therapeutic efficacy.

## 6 Targeting ferroptosis for the prevention and treatment of neurodegenerative diseases: strategies and future perspectives

Ferroptosis, an iron-dependent form of regulated cell death driven by lipid peroxidation, has emerged as a critical mechanism in the pathogenesis of various neurodegenerative diseases and aging-related disorders (404–408). The identification of ferroptosis in disease models is increasingly recognized as a key research direction for elucidating disease mechanisms and developing novel therapeutic strategies. Currently, the association between ferroptosis and disease progression is primarily investigated through three experimental approaches: i) genetic manipulation: Knockout or knockdown of core ferroptosis regulators—such as *GPX4*, *SLC7A11*, and *ACSL4*—is used to assess resulting pathological changes. ii) Biomarker detection: Levels of lipid peroxidation products—such as MDA—are measured in human tissues or animal models to evaluate ferroptotic activity. iii) Pharmacological intervention: The efficacy of ferroptosis inhibitors such as Fer-1, liproxstatin-1, and DFO is tested in preclinical models to determine their neuroprotective potential. These methodologies collectively provide a robust foundation for targeting ferroptosis in both basic research and translational medicine.

### 6.1 Ferroptosis as a therapeutic target in neurodegeneration

With growing interest in ferroptosis across various degenerative diseases and organ injuries, numerous inhibitors have been identified that effectively slow disease progression under different pathological conditions, with several advancing to preclinical or clinical investigation. Ferroptosis is primarily suppressed through three strategies: chelating iron ions to block the Fenton reaction, preventing lipid peroxidation, and eliminating lipid peroxides. Among these approaches, iron chelators and free radical scavengers are recognized as key inhibitors of ferroptosis.

Clinically used iron chelators such as DFO and DFP, initially developed for the treatment of iron overload in β-thalassemia and hemochromatosis, have been shown to mitigate tissue injury by inhibiting Fenton reaction-driven lipid peroxidation (409, 410). In addition, deferasirox (DXZ) has demonstrated protective effects against doxorubicin-induced cardiotoxicity by inhibiting ferroptosis (411). Given iron’s pivotal role in ferroptotic cell death and neurodegeneration, the therapeutic potential of iron chelators has been extended to conditions such as AD and PD. CN128, a novel iron chelator approved for the treatment of iron overload in thalassemia and related disorders, has also shown experimental efficacy in models of PD (412).

Another widely studied class of ferroptosis inhibitors includes free radical scavengers, exemplified by Fer-1. As one of the first compounds specifically identified to inhibit ferroptosis, Fer-1 effectively prevents cell death in multiple *in vitro* models and reduces tissue injury in animal studies (413). Structural optimization has led to the development of second- and third-generation ferrostatins, such as liproxstatin-1, SRS11-92, SRS16-86, and UAMC-3203, which exhibit enhanced stability and *in vivo* activity (414, 415). Additionally, conventional antioxidants such as α-tocopherol (vitamin E), NAC, and butylated hydroxytoluene (BHT) have shown similar radical-scavenging capabilities and can inhibit ferroptosis in a manner comparable to ferrostatins and liproxstatins (416).

### 6.2 Clinical translation of ferroptosis inhibition in neurodegenerative diseases

With increasing evidence linking ferroptosis to neurodegeneration, targeting this pathway holds significant promise for drug discovery and precision therapy. Emerging studies indicate that ferroptosis not only affects neuronal viability but also influences glial function and immune response dynamics, offering new opportunities to modulate disease progression through immunometabolic regulation (404–408). In AD and PD, interventions aimed at suppressing ferroptosis—such as iron chelation, GSH replenishment, and GPX4 activation—have shown beneficial effects in preclinical models, including reduced oxidative stress, improved cognitive and motor functions, and delayed disease onset (409–412). However, these findings must be validated in clinical trials to ensure safety, efficacy, and BBB permeability. Advances in structural biology—particularly the integration of AlphaFold and RoseTTAFold—have enabled rational drug design targeting key ferroptosis effectors such as ACSL4, LOXs, and system Xc^−^ components. Combined with high-throughput screening, these tools facilitate the discovery of novel small-molecule probes and inhibitors with enhanced specificity and potency. Moreover, the application of multi-omics and single-cell technologies in neurodegenerative disease research allows for spatial and temporal resolution of ferroptosis across distinct cell types. This enables the identification of disease-specific regulatory pathways and biomarkers, laying the groundwork for targeted therapeutics and personalized treatment strategies. Despite promising preclinical data, several challenges remain before ferroptosis-targeted therapies can be translated into clinical practice: i) clinical relevance: Most current evidence derives from cell culture and animal models. Whether ferroptosis contributes directly to human neurodegenerative pathology remains uncertain (404–408). Longitudinal studies assessing lipid peroxidation markers, iron levels, and GPX4 expression in patient cohorts are essential to validate ferroptosis as a driver of neuronal loss. ii) Biomarker development: A lack of specific and reliable biomarkers limits the ability to monitor ferroptosis in clinical settings. Current methods rely on indirect measures such as MDA, 4-HNE, and ferritin levels, which may not be sufficient to distinguish ferroptosis from other forms of oxidative stress-driven cell death. Therefore, identifying direct markers of ferroptosis—such as oxidized phospholipids or GPX4 degradation—is crucial for clinical diagnosis and therapeutic monitoring. iii) Context-dependent roles: The functional role of ferroptosis may vary depending on disease stage and tissue context. For example, in hepatocellular carcinoma (HCC), ferroptosis serves as a tumor-suppressive mechanism in early stages but becomes dysregulated in advanced disease, suggesting that dynamic modulation may be necessary during different phases of neurodegeneration. Understanding the dualistic nature of ferroptosis will be vital for designing safe and effective interventions. i) Drug delivery and specificity: Many ferroptosis inhibitors exhibit limited brain penetration and off-target effects. Enhancing CNS bioavailability through nanoparticle formulations, prodrug strategies, or intranasal delivery systems could improve therapeutic outcomes. ii) Mechanistic gaps: While numerous proteins and pathways have been implicated in ferroptosis, many studies focus on upstream events. The downstream execution machinery of ferroptosis—and whether a single terminal effector exists—remains incompletely understood. Further mechanistic dissection is required to identify actionable targets along the entire ferroptotic cascade.

### 6.3 Broad clinical potential of targeting ferroptosis in neurodegeneration

**Figure 10:**
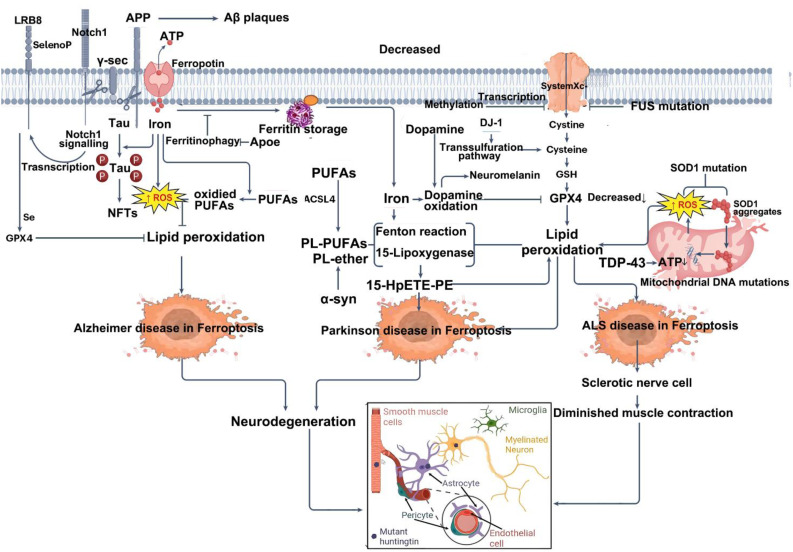
A common ferroptosis network in neurodegenerative diseases.

Targeting ferroptosis offers a novel approach to neuroprotection by preserving cellular integrity, modulating inflammation, and enhancing antioxidant defenses. Given its central role in determining the fate of neurons and glial cells, ferroptosis inhibition could reshape the therapeutic landscape of neurodegenerative diseases. Several lines of evidence suggest that manipulating ferroptosis can yield favorable outcomes: i) Iron chelators reduce iron-mediated oxidative stress and preserve mitochondrial function. ii) Antioxidants and radical scavengers counteract lipid ROS and prevent membrane destruction. iii) Regulation of microglial polarization via ferroptosis-sensitive pathways may shift the inflammatory milieu toward neuroprotection. However, successful clinical translation requires rigorous validation of molecular targets, optimization of pharmacokinetics, and comprehensive evaluation of long-term safety. The integration of AI-driven drug discovery, advanced imaging techniques, and multimodal omics analysis will accelerate the development of selective and potent ferroptosis inhibitors tailored to specific disease contexts. Ultimately, targeting ferroptosis may provide a unified framework for understanding and treating neurodegenerative diseases. As our knowledge expands, so too does the opportunity to harness this pathway for therapeutic benefit, potentially offering new hope for millions affected by currently incurable neurological disorders (**Figure 10**).

## 7 Limitations and future directions of ferroptosis in neurodegenerative diseases

Although mounting evidence highlights the critical role of ferroptosis in the onset and progression of neurodegenerative diseases, and its status as a “novel mode of cell death” is increasingly recognized, the field remains at an early stage of conceptual integration and mechanistic exploration. Within the complex disease context, fundamental questions remain regarding the specificity, detectability, and spatiotemporal dynamics of ferroptosis, as well as its interactions with classical pathological pathways such as Aβ, tau, α-synuclein, and mutant huntingtin protein. These unresolved issues not only constrain its translational potential as a therapeutic target but also challenge our understanding of the nature of neurodegenerative diseases.

A major limitation lies in the absence of reliable, neuron-specific biomarkers of ferroptosis. Current detection methods mainly rely on indicators in cell or animal models, such as lipid peroxidation products, iron content, GPX4 activity, or ACSL4 expression. However, no highly specific and reproducible markers have yet been established in human brain tissue or biofluids. The complexity of pathology, cellular heterogeneity, and stage-dependent changes in disease progression make it difficult to disentangle ferroptosis-specific signals from overlapping oxidative stress responses. To advance clinical application, future research should develop multimodal biomarker panels by integrating technologies such as mass spectrometry imaging, spatial transcriptomics, and single-cell lipidomics, combined with artificial intelligence to define a distinct “ferroptosis signature” in neurodegeneration.

Another unresolved issue concerns the bidirectional relationship between ferroptosis and pathological proteins. Evidence suggests that Aβ can enhance neuronal iron uptake and promote GPX4 degradation, while lipid peroxidation products may accelerate tau hyperphosphorylation. Similarly, α-synuclein aggregation can suppress system Xc^−^ activity, leading to glutathione depletion, whereas membrane damage caused by ferroptosis may in turn facilitate the intercellular spread of α-synuclein. Mutant huntingtin protein disrupts iron metabolism-related gene expression and can be stabilized in its toxic conformation by lipid peroxides. Yet, it remains unclear whether ferroptosis serves primarily as a downstream effector, an upstream driver, or part of a self-amplifying feedback loop in disease progression. Addressing this question will require conditional genetic models and *in vivo* imaging approaches to clarify causal interactions across different stages, brain regions, and cell types.

The unique characteristics of central iron homeostasis and the presence of the BBB also pose therapeutic challenges. Iron turnover in the brain is tightly regulated by transferrin receptors, ferritin, ferroportin, and neuron–glia interactions. However, most ferroptosis inhibitors show poor BBB penetration, often necessitating high systemic doses or direct intracerebral administration in animal studies, limiting their clinical applicability. Iron chelators, while capable of reducing brain iron, risk systemic side effects by failing to discriminate between pathological iron accumulation and physiological iron requirements. Moreover, glial cells such as microglia and astrocytes play a dual role in buffering iron: while they can sequester iron released from dying neurons, their own ferroptosis may exacerbate inflammation and oxidative stress. Future therapeutic strategies should therefore focus on brain-targeted delivery systems, cell type-specific modulators of iron metabolism, and approaches aimed at rebalancing iron cycling rather than simple chelation.

Equally important is the close interplay between ferroptosis and neuroinflammation. Neurons undergoing ferroptosis release damage-associated molecular patterns (DAMPs) such as HMGB1, ATP, and lipid peroxidation products, which activate microglia and drive them toward a pro-inflammatory phenotype. This in turn disrupts the BBB, recruits peripheral immune cells, and induces further ferroptosis, creating a vicious cycle of “ferroptosis–inflammation–ferroptosis.” Whether this process functions as an inflammation amplifier in specific brain regions, and whether ferroptosis inhibition alone can mitigate inflammation or must be combined with anti-inflammatory therapies, remains unclear. Future studies employing real-time *in vivo* imaging and single-cell immune profiling will be essential for mapping the interactions between ferroptotic cells and immune populations and for identifying key nodes along the ferroptosis–inflammation axis as therapeutic targets.

Despite encouraging findings in animal models, the translation of ferroptosis inhibitors into clinical application remains a formidable challenge. Barriers include the lack of well-defined therapeutic windows, the difficulty of attributing neuroprotective effects of natural compounds to ferroptosis specifically, and the absence of biomarker-guided clinical trial designs. Moving forward, it will be necessary to establish early screening frameworks based on ferroptosis risk scoring, which could integrate genetic susceptibility, iron metabolism polymorphisms, neuroimaging of iron deposition, and plasma lipid peroxidation markers. Parallel efforts should prioritize the development of next-generation ferroptosis inhibitors that are mechanistically precise, brain-targeted, and orally bioavailable, along with the design of biomarker-driven clinical trials to test disease-modifying efficacy in patients with AD, PD, and HD.

In summary, the concept of ferroptosis has opened a new dimension for understanding neuronal cell death and offers unprecedented opportunities for therapeutic intervention. Yet, the complexity of the nervous system, the chronic progression of disease, the diversity of pathological proteins, and the restrictive nature of the BBB collectively constitute formidable obstacles. Future research must move beyond the simplistic paradigm of “inhibition equals benefit” toward spatiotemporally precise, cell type-specific, pathway-integrated, and biomarker-guided strategies. Only then can ferroptosis evolve from a mechanistic hotspot in basic research to a true clinical breakthrough, bringing tangible hope to patients with neurodegenerative diseases.
